# Near-Infrared Luminescence from Transparent Thin Films of Copper(I) Thiocyanate Modified with 2‑Mercaptobenzothiazole via Excited-State Symmetry Breaking

**DOI:** 10.1021/acs.inorgchem.5c05480

**Published:** 2026-04-09

**Authors:** Saran Waiprasoet, Paul A. Hume, James M. Scott, Phattananawee Nalaoh, Pongkamon Prayongkul, Daniel M. Packwood, Justin M. Hodgkiss, David J. Harding, Pichaya Pattanasattayavong

**Affiliations:** † Department of Materials Science and Engineering, School of Molecular Science and Engineering, Vidyasirimedhi Institute of Science and Technology (VISTEC), Rayong 21210, Thailand; ‡ MacDiarmid Institute for Advanced Materials and Nanotechnology, Wellington 6012, New Zealand; § The Dodd-Walls Centre for Photonic and Quantum Technologies, Dunedin 9016, New Zealand; ∥ School of Chemical and Physical Sciences, Victoria University of Wellington, Wellington 6012, New Zealand; ⊥ Institute for Integrated Cell-Material Sciences (iCeMS), Kyoto University, Kyoto 606-8317, Japan; # School of Chemistry, Institute of Science, 65162 Suranaree University of Technology, Nakhon Ratchasima 30000, Thailand

## Abstract

Near-infrared (NIR) emission in molecular materials is typically achieved by narrowing the band gap through extended π-conjugation or heavy-atom substitution. Here, we report an unconventional route—excited-state symmetry breaking—that enables NIR emission while preserving a wide band gap. Copper­(I) thiocyanate (CuSCN), a transparent semiconductor with optical activity normally observed only in the ultraviolet region, forms a dimeric complex with 2-mercaptobenzothiazole (MBTz). In thin films, CuSCN-MBTz complex exhibits NIR photoluminescence at 800 nm, while retaining a large optical gap (>3 eV), resulting in a transparent NIR-emissive material with a large Stokes shift of ∼15,000 cm^–1^. Structural and spectroscopic analyses reveal that, unlike the rigid crystalline phase, the film state allows molecular reorganization of Cu_2_(SCN)_2_(MBTz)_4_ dimers, which induces symmetry breaking and lowers the energy of the emissive state without reducing the band gap, in contrast to typical Cu­(I) emitters. This work provides a new photophysical mechanism for generating long-wavelength emission from wide-band gap, earth-abundant Cu­(I) systems and widens the functional scope of CuSCN materials.

## Graphical abstract

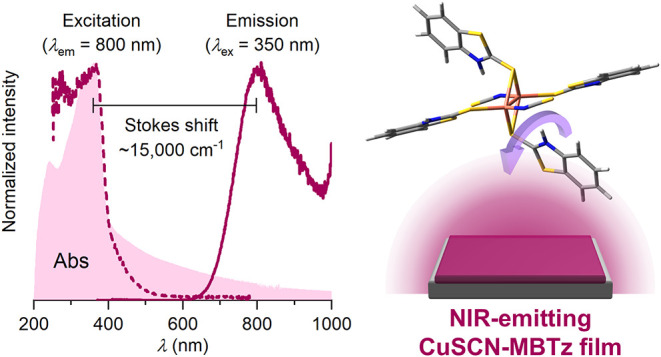

## Introduction

The demand for near-infrared (NIR) emissive materials is growing rapidly due to a myriad of applications, such as organic light-emitting diodes (OLEDs), biological/medical imaging, night-vision readable displays, and security applications among others.^1–3^ While standard NIR emitters are usually complexes of precious or rare-earth metals,^4–6^ Cu­(I) complexes are emerging as promising candidates for NIR luminescent materials due to their material sustainability and cost-effectiveness.^7–11^ One of the Cu­(I)-based functional materials is copper­(I) thiocyanate (CuSCN), a *transparent* hole-transporting coordination polymer (CP) semiconductor. CuSCN has captured attention in coordination chemistry and materials science due to its well-demonstrated potential in opto/electronic applications and continuous device developments.^12–16^ The extensive utilization of CuSCN is underpinned almost entirely by its excellent optical transparency, resulting from its wide band gap. However, its structural versatility allows for various coordination networks with a multitude of coligands (L), enabling the fine-tuning of its electronic and optical properties.^17^ As shown in this work, we can employ CuSCN as a material platform to generate NIR emission out of a transparent thin film.

Early on, Pike et al. demonstrated how the introduction of amine and imine ligands alters the 3D CuSCN structure and yields some CPs with luminescent behavior.^18^ Contrary to the transparent and nonemissive CuSCN (weak emission in the range 350–400 nm or 3.11–3.55 eV sometimes reported), complexes between CuSCN and substituted pyridine (Py) ligands exhibited tunable photoluminescence (PL) from blue to orange. Several of the complexes displayed high photoluminescence quantum yield (PLQY) of 31 to 66%, mostly emitting in the green to yellow region, with peak wavelengths (λ_PL,max_) between 480–530 nm (2.35–2.59 eV). The longest λ_PL,max_ at 585 nm (2.12 eV) was obtained from a complex between CuSCN and quinoline (CuSCN:L ratio of 1:2), which also showed a large Stokes shift of 10,840 cm^–1^ but low PLQY of <3%. Recently, we also reported a systematic tuning of the energy levels in 2D CPs of (1:1) CuSCN-L where L is a series of 3-substituted Py (3-XPy, where X = OMe, H, Br, or Cl).^19^ With the increasing electron-withdrawing ability of the substituent, the conduction band minimum (CBM) was decreased and the emission red-shifted. However, λ_PL,max_ was only around 500 nm (∼2.50 eV). Artem’ev et al. reported monomeric CuSCN-L complexes based on tris­(2-pyridyl)­phosphine oxide (Py_3_PO) as a coligand.^20^ Mono- and dinuclear complexes [Cu­(NCS)­(Py_3_PO) and Cu_2_(NCS)_2_(Py_3_PO)_2_] exhibited PL at 610 nm (2.04 eV) whereas a trinuclear complex [Cu_3_(NCS)_3_(Py_3_PO)_2_] showed a slightly blue-shifted emission at 590 nm (2.11 eV). Nevertheless, their PLQY was also low, ranging between 0.5 to 3%. Interestingly, CuSCN coordinated with tris­(2-pyridyl)­phosphine (Py_3_P) was found to be a 1D [Cu­(NCS)­(Py_3_P)]*_n_* polymer, in which CuNCS units are bridged by Py_3_P. Despite the strong intensity and a PLQY of 10%, the emission was shifted to green with λ_PL,max_ of 510 nm (2.44 eV).

These studies highlight that while luminescence from CuSCN-L complexes can be tuned across the visible range, achieving NIR emission has remained elusive. For narrow-band emitters with nested potential energy surfaces, reducing the optical gap often enhances nonradiative decay, in accordance with the energy-gap law.^21^ For broad-band emitters with strong vibronic coupling, as is the case for Cu­(I) complexes, lowering the energy of the emissive state toward NIR can also decrease the thermal activation crossover barriers (smaller reorganization energies), thus leading to a higher probability for nonradiative relaxation.^22^ Sulfur-rich ligands can partially offset this by stabilizing the excited state through π-S conjugation,^23,24^ as shown in benzothiazole-containing Cu­(I) complexes that shift the emission toward red or NIR regions.^7^ However, such emission still arises from reduced band gaps, which compromises transparency and limits device integration.

Here, we demonstrate a fundamentally different route: a *transparent* CuSCN-based thin film that emits strongly in the NIR. Using simple thiazole-based coligands, including thiazole (Tz), benzothiazole (BTz), 2-mercaptothiazole (MTz), and 2-mercaptobenzothiazole (MBTz), we discovered that CuSCN-MBTz uniquely exhibits intense NIR emission with λ_PL,max_ of 800 nm (1.55 eV) when prepared as a thin film while retaining a large optical band gap (>3 eV). This emission is accompanied by an exceptionally large Stokes shift of ∼15,000 cm^–1^ and a relatively high PLQY of 23%. Our detailed experimental and theoretical studies show that the NIR emission originates from excited-state symmetry breaking and molecular relaxation in the film with low crystallinity, representing a rare case of wide-gap NIR luminescence. This mechanism transforms a common transparent interlayer material into a new class of optically active coordination semiconductors. We note that a preprint version of this manuscript has been deposited to ChemRxiv.^25^

## Results and Discussion

To prepare the CuSCN-L complexes, CuSCN was first fully dissolved in diethyl sulfide (DES). Then, the four selected thiazole-based coligands (Tz, BTz, MTz, and MBTz) were each added to separate vials of the prepared CuSCN solution. The CuSCN:L molar ratio was varied from 2:1, 1:1, and 1:2 with a fixed total concentration of 0.15 M. Slow evaporation led to crystallization of the products, with selected crystals collected for structural determination by single-crystal X-ray diffraction (SC-XRD). To prepare bulk powder samples, the collected crystals were thoroughly washed with ethanol to remove any unreacted coligands. After drying, the washed crystals were ground manually for further characterizations by power X-ray diffraction (PXRD), photoelectron yield spectroscopy (PYS), Raman spectroscopy, thermogravimetric analysis (TGA), ultraviolet–visible spectroscopy (UV–vis), and photoluminescence spectroscopy (PL). Full details are described in the Methods Section.

### Structural Characterizations and Thermal Stability

Hereinafter, complexes of CuSCN with Tz, BTz, MTz and MBTz coligands are designated as CuSCN-Tz, CuSCN-BTz, CuSCN-MTz and CuSCN-MBTz, respectively. Crystal structures determined by SC-XRD (CCDC 2487614, 2487615, 2487616, and 2487617) are shown in Figure 1. Crystallographic data are reported in Table S1. The complexes with thiazole rings (Tz and MTz) yield a CuSCN:L ratio of 1:1 whereas the complexes with the benzene-fused analogs (BTz and MBTz) result in a CuSCN:L ratio of 1:2. Tz and BTz coligands coordinate the Cu­(I) centers with the nitrogen atom of the thiazole ring while the other two, MTz and MBTz, coordinate with the sulfur atom of the mercapto group. For the latter, both coligands exist as the thione tautomer in which the thiazole nitrogen is protonated.

**Figure 1:**
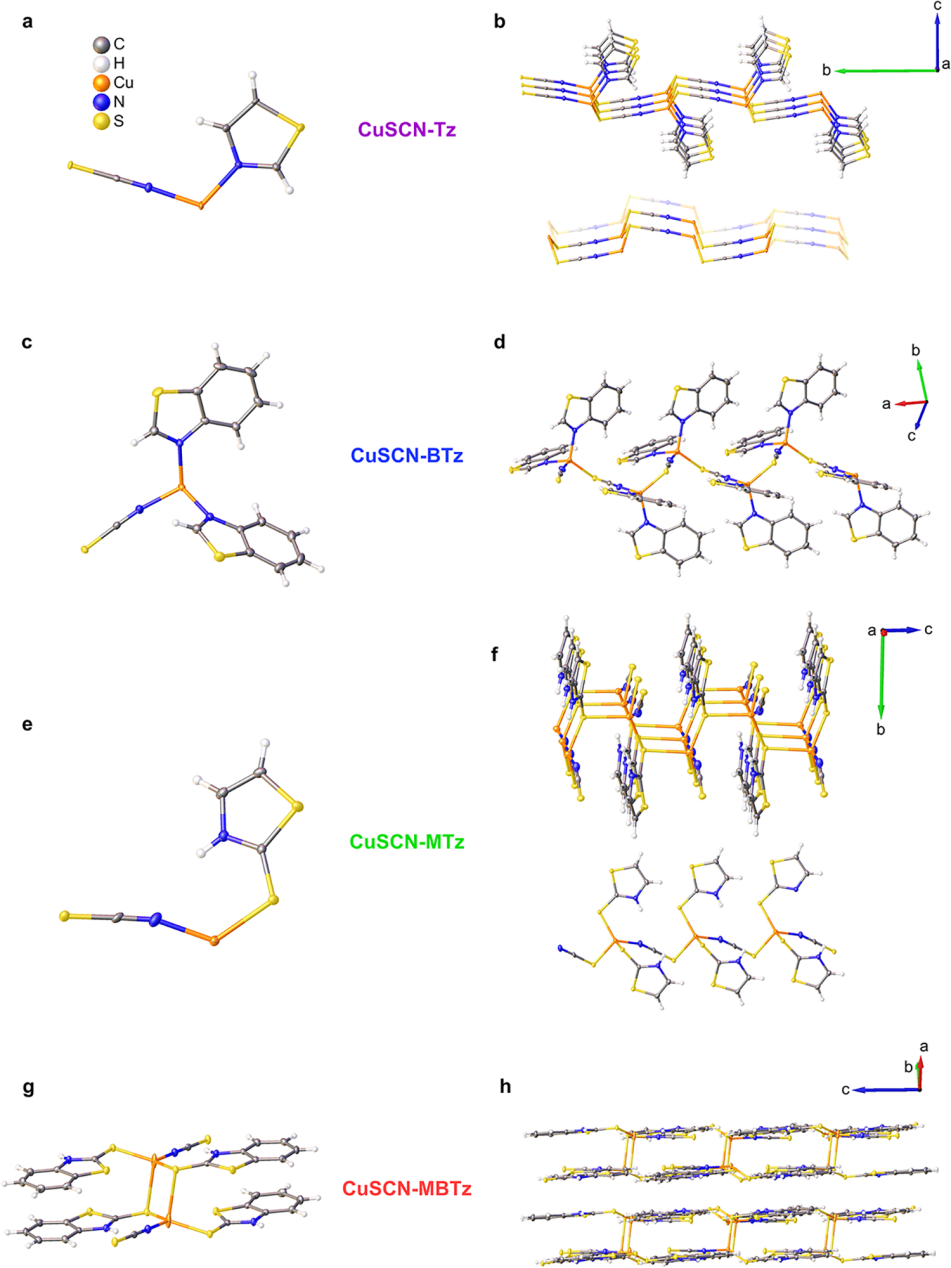
Asymmetric units and extended structures of CuSCN complexes with thiazole-based ligands. (a, b) CuSCN-Tz ([Cu­(SCN)­(Tz)]*_n_*) forms 2D sheets stacked along the *c*-axis with the Cu-SCN network showing a hexagonal-like motif in a boat-like conformation. (c, d) CuSCN-BTz ([Cu­(SCN)­(BTz)_2_]*_n_*) forms 1D helical chains. (e, f) CuSCN-MTz ([Cu­(SCN)­(MTz)]*_n_*) forms 2D sheets via bridged 1D zigzag chains, stacked along the *b*-axis. (g, h) CuSCN-MBTz forms a dimeric unit, Cu_2_(SCN)_2_(MBTz)_4_ with a Cu_2_S_2_ core. Thermal ellipsoids are drawn at 50% probability.

For CuSCN-Tz, the resulting structure is a 2D CP with a formula [Cu­(SCN)­(Tz)]*_n_* (Figure 1a,b). The sulfur atoms of the SCN ligand bridge pairs of Cu atoms in this 2D sheet structure. In our previous report, complexes of CuSCN with 3-OMePy and Py (also 1:1 ratio) crystallize in a structure resembling 2D silicene/germanene in the chair-like conformation with the coligands all coordinating on one side of the layer.^19^ In the case of CuSCN-Tz, the 2D Cu-SCN network resembles a boat-like conformation (Figure 1b, bottom) with Tz coligands coordinating on both the top and bottom sides of the 2D layer. The 2D sheets stack along the *c*-axis with an interlayer distance of 11.89 Å. For CuSCN-BTz with a formula [Cu­(SCN)­(BTz)_2_]*_n_*, the structure was found to consist of 1D helical chains due to the steric effect of the bulky coligand, with the SCN ligands bridging the Cu centers (Figure 1c,d). CuSCN-MTz, having a formula [Cu­(SCN)­(MTz)]*_n_*, was obtained as 2D sheets that are formed by 1D zigzag chains of Cu atoms bridged by the SCN ligands (Figure 1e,f). Additional bridging between the Cu centers of adjacent chains involving the sulfur atoms (mercapto group) of the coligands creates the 2D sheets. However, the topology is different from that of CuSCN-Tz, having no hexagonal-like motifs. The 2D structures are stacked along the *b*-axis with an interlayer distance of 10.60 Å. In contrast, CuSCN-MBTz did not yield a polymeric network but a dimeric unit Cu_2_(SCN)_2_(MBTz)_4_ with a Cu_2_S_2_ diamond core and π–π interactions between the coligands within the dimer (Figure 1g,h). The SCN ligands are terminal and *N*-bound in this structure. Two nearly equivalent Cu···Cu contacts are present within the molecular structure, with separations of 3.16 and 3.33 Å. No significant contacts between adjacent dimer units exist as the shortest intermolecular Cu–Cu distance is relatively large at 4.14 Å. Likewise, the MBTz ligands do not show any significant intermolecular interactions between neighboring dimers as they are stacked offset from each other.

All Cu­(I) centers in the four complexes are 4-coordinate, and the geometry index *T*_4_ can be calculated from

1 $$T_{4}=\frac{360^{{}^{\circ}}-\left(A_{\alpha }+A_{\beta }\right)}{141^{{}^{\circ}}}$$

where *A*_α_ and *A*_β_ are the two largest angles around the 4-coordinate Cu­(I) centers.^26^ A *T*_4_ value of 1 represents a perfect tetrahedral geometry. CuSCN-Tz shows the highest *T*_4_ of 0.96, displaying a nearly ideal tetrahedral geometry. CuSCN-BTz and CuSCN-MTz exhibit moderate distortion with *T*_4_ ≈ 0.90 due to steric effects from bulky or bridging ligands. CuSCN-MBTz presents the most distorted tetrahedral geometry with *T*_4_ ≈ 0.82 and 0.88 for the two Cu­(I) centers, reflecting strong structural constraints in the crystalline packing. As shown later, CuSCN-MBTz undergoes significant structural reorganization when unrestricted by the crystal structure.

Thermogravimetric analysis (TGA) was performed on bulk powder samples under N_2_ to study their thermal stability. For reference, CuSCN decomposes between 410 and 440 °C as shown in Figure S1. TGA results of CuSCN-Tz, CuSCN-BTz, CuSCN-MTz, and CuSCN-MBTz complexes showed a first mass loss around 115, 125, 220, and 230 °C, respectively, followed by a second mass loss around 400–410 °C as displayed in Figure S2. The latter agrees well with the decomposition of CuSCN, hence indicating that the first mass loss can be attributed to the loss of the heterocyclic coligands. Complexes with a fused benzene ring (BTz and MBTz) decomposed at slightly higher temperatures (125 and 230 °C) compared to simple thiazole rings (Tz and MTz) (115 and 220 °C), consistent with additional dispersion interactions from the benzene rings. The mercapto-based complexes (MTz, MBTz) exhibited ≈100 °C higher stability, confirming that Cu–S coordination is considerably stronger than Cu–N binding.

### Photophysical Properties of the Complexes in Bulk Powder Form

Photophysical properties of the complexes in the bulk structure (as ground powders) were characterized by using ultraviolet–visible spectroscopy (UV–vis), photoluminescence spectroscopy (PL), photoluminescence excitation spectroscopy (PLE), and time-resolved photoluminescence spectroscopy (TRPL). The structure of CuSCN features a 2D extended network of Cu–S bonds which form a robust and dispersed valence band (VB), favorable for hole transport.^27^ The 2D Cu–S sheets are separated by C≡N (part of the linear SCN ligand), of which the π* states form the high-energy conduction band (CB), resulting in a large band gap of >3.5 eV and high transparency.^28–30^ All CuSCN–L complexes exhibited broad UV absorption extending into the violet–blue region (Figure 2). Notably, the absorption edge of CuSCN-MBTz was around 510 nm, giving it a yellow color in appearance. As expected, the coordination with aromatic coligands significantly red-shifts the absorption of the complexes through the lowering of the CBM or lowest unoccupied molecular orbital (LUMO) energy levels. The excited state of CuSCN-L complexes is well-known to have a metal-to-ligand charge transfer (MLCT) character.^17–19^ The peaks associated with the maximum absorption are reported in Table 1. The presence of the fused benzene ring and the mercapto group further stabilizes the MLCT state (lowering the energy).^7^

**Figure 2:**
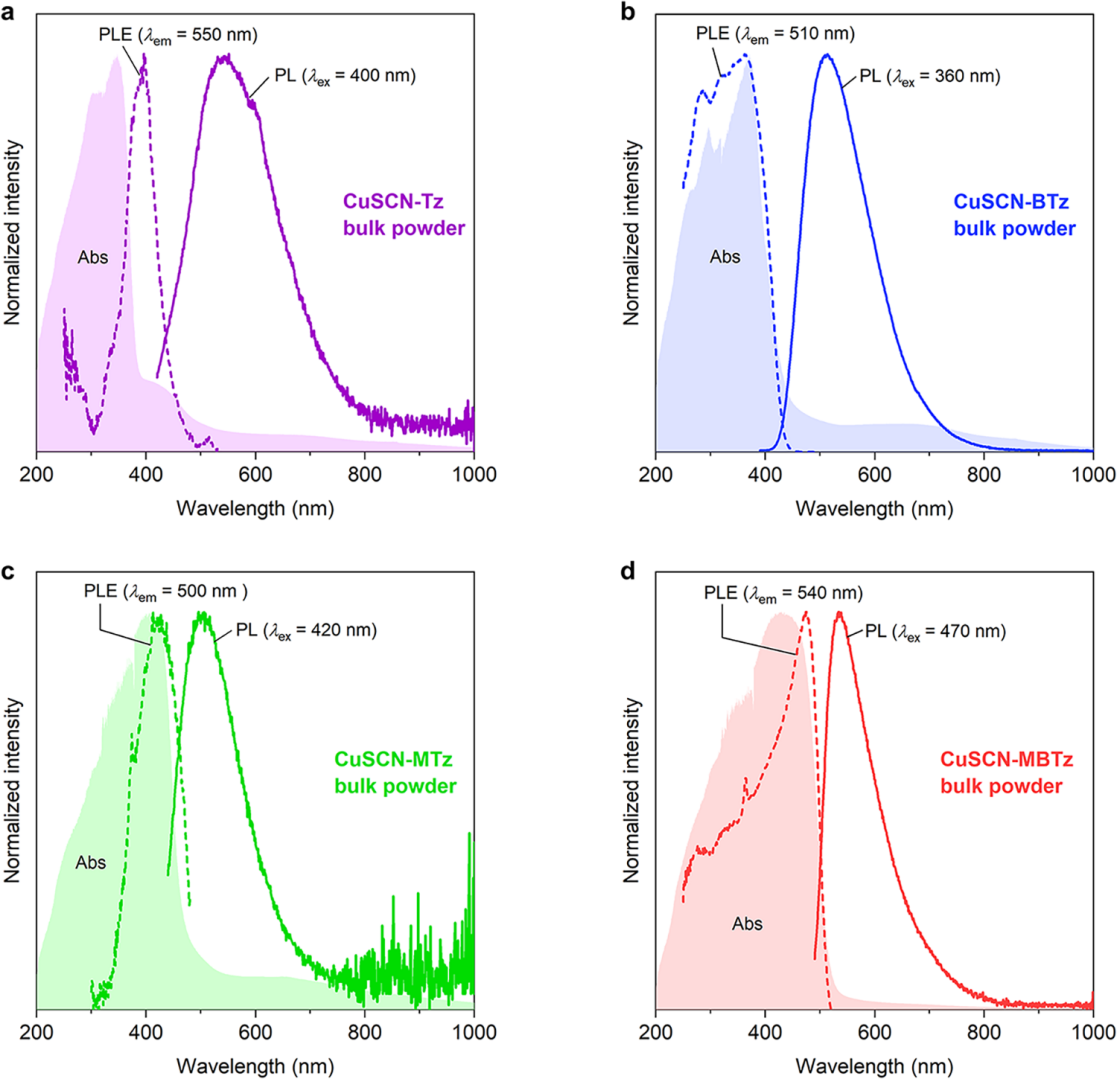
Absorption (Abs), photoluminescence (PL) spectra, and photoluminescence excitation (PLE) spectra of (a) CuSCN-Tz, (b) CuSCN-BTz, (c) CuSCN-MTz, and (d) CuSCN-MBTz in bulk powder form. Excitation wavelengths (λ_ex_) and emission wavelengths (λ_em_) for PL and PLE measurements, respectively, are annotated in the plots.

**Table 1:** Photophysical Properties of CuSCN-L Complexes in Bulk Powder form

**complex**	**absorption** **λ**_**abs,max**_ **(nm)**	**excitation** **λ**_**PLE,max**_ **(nm)**	**emission** **λ**_**PL,max**_ **(nm)**	**PLQY Φ (%)**	**PL lifetime** **⟨τ⟩**_**int**_ **(μs)**	**PL fwhm** (cm^–**1**^ **)**	**absorption–emission Stokes shift** (cm^–**1**^ **)**	excitation–emission Stokes shift (cm^–**1**^ **)**
CuSCN-Tz	346	394	545	n/a^a^	9.1	6262	10,553	7032
CuSCN-BTz	370	364	512	22.6	6.0	4978	7496	7941
CuSCN-MTz	407	424	502	n/a^a^	4.3	5166	4650	3665
CuSCN-MBTz	434	473	535	n/a^a^	5.7	3409	4350	2450

a PLQY of CuSCN-Tz, CuSCN-MTz, and CuSCN-MBTz in the bulk powder form was below the instrument limit and could not be measured quantitatively.

Figure 2 also shows the PLE and PL spectra. We observe that the PLE spectra match well with the absorption edge of the UV–vis measurements, confirming that the luminescence is derived from the MLCT state. Note that for CuSCN-Tz, the PLE peak still coincides with a prominent shoulder in the UV–vis absorption (around 400–420 nm), also associated with MLCT. The higher-energy absorption in this case can be assigned to intraligand or ligand-centered (LC) transitions.^7,31,32^ All complexes in the bulk structure luminesced in the 500–550 nm range (green), a typical region for many CuSCN-L complexes with simple aromatic coligands.^18,19^ From TRPL, the emission decay followed a biexponential model as shown in Figure S3a–d with the fitting parameters reported in Table S2. The intensity-average lifetimes (⟨τ⟩_int_) accounting for the relative contribution of each emissive pathway (see Table 1) were found to be in the μs time scale for all complexes. While the emission profiles closely resemble mirror images of the absorption/excitation spectra and the Stokes shifts are on the same order as the full width at half-maximum (fwhm) values, suggesting fluorescence, the μs time scale indicates that they more likely originate from thermally activated delayed fluorescence (TADF). Indeed, due to the relatively strong spin–orbit coupling (SOC) of Cu, many Cu­(I) complexes are known to exhibit TADF,^33^ especially those with a strong MLCT character that reduces the energy difference between the singlet and triplet states. As shown further below, temperature-dependent PL and TRPL data of CuSCN-MBTz also show strong evidence for the TADF mechanism. We note also that complexes with N-coordinating ligands (CuSCN-Tz and CuSCN-BTz) displayed larger Stokes shifts than those with S-coordinating ligands (CuSCN-MTz and CuSCN-MBTz), suggesting more structural reorganization upon excitation for complexes in which the aromatic rings coordinate directly to the metal. In addition, for the structures which lack the mercapto spacer, the electrons are expected to be spatially closer in the excited state following MLCT. The resulting larger exchange energies could lower the triplet energies, inferring relatively larger singlet–triplet energy differences compared to the other complexes with the mercapto spacer unit. This is highlighted in the case of CuSCN-Tz which has the smallest ligand and no mercapto group; it was found to exhibit the longest PL lifetime and broadest PL fwhm, indicating some triplet contribution mixed in the emission due to the larger singlet–triplet energy gap.

Interestingly, CuSCN-BTz exhibited a relatively high PLQY of 22.6% whereas for the other three complexes, the yield was below the instrumental limit, hence prohibiting accurate measurements. As the PL lifetimes were on the same order, the high PLQY of CuSCN-BTz was possibly resulting from a larger radiative decay rate. For the three network structures, CuSCN-BTz exists in the 1D structure whereas CuSCN-Tz and CuSCN-MTz have 2D dimensionality. Our previous study on electronic structures of various CuSCN-*co*-ligand complexes has found that charge carriers are significantly more localized in 1D structures compared to 2D.^17^ It could be inferred then that the recombination probability is higher in 1D CuSCN-BTz, hence a higher apparent radiative decay rate and PLQY. As for CuSCN-MBTz, we surmise that enhanced intramolecular π–π interactions of MBTz within the dimeric unit and intermolecular interactions among the dimers could lead to more nonradiative deactivation pathways, reducing PLQY in the bulk form. As shown further below, these factors are weakened in the thin-film and molecular state, thus increasing PLQY.

### Thin-Film Formation and NIR Emission from CuSCN-MBTz Film

As CuSCN is one of the few CPs that can be solution-processed into thin films, we applied this unique processing route on the complexes herein. CuSCN-L complexes were dissolved in DES and spin-coated onto substrates to obtain thin-film samples (see Methods Section for details). Powder X-ray diffraction (PXRD) was used to investigate the crystalline phases and structural properties, with the results shown in Figure S4. Film samples were measured with a grazing incidence (GIXRD) setup. The reference CuSCN film exhibited the usual diffraction pattern and can be identified as the 3R β-CuSCN phase with diffraction peaks at 2θ = 16.2, 27.3, and 32.7° associated with the (0 0 6), (1 0 2̅), and (0 0 12) planes, respectively.^34,35^ For CuSCN-Tz and CuSCN-BTz, the resulting films completely reverted to the structure of the parent CuSCN (Figure S4a,b), suggesting that these complexes are not sufficiently robust to undergo spin-coating. This result corroborates well with our discussion in the previous section that the interactions between Cu and thiazole N are relatively weak. In contrast, CuSCN-MTz and CuSCN-MBTz films preserved some characteristics of the bulk powder (Figure S4c,d). We note that for CuSCN-MBTz, the XRD pattern of the powder sample showed some differences to the simulated pattern based on single-crystal data, likely due to grinding effects. Compared to the other three complexes that feature network structures, the crystal of CuSCN-MBTz packs via intermolecular interactions between the molecular dimers, hence it is more susceptible to external mechanical forces. A closer inspection of CuSCN-MBTz powder and film samples (Figure S4e) shows that the latter retained the overall pattern, but the peaks were broadened and shifted to lower diffraction angles (wider *d*-spacings). This suggests a more loosely packed structure for thin films of CuSCN-MBTz which is crucial for the unique photophysical properties, as discussed further below.

Photophysical properties of thin films were also investigated by UV–vis, PL, PLE, and TRPL, similar to the study of the bulk. The UV–vis absorption spectrum of a control CuSCN film (also spin-coated from DES solution) was recorded as a reference. For CuSCN-Tz and CuSCN-BTz (Figure S5a,b), their absorption spectra only displayed characteristics of CuSCN, i.e., a shoulder around 300 nm and an intense peak around 240 nm associated with Cu 3d–SCN π* and SCN π–π* electronic transitions, respectively.^17,36^ This is in accordance with the XRD results, proving that the coligands Tz and BTz were lost following solution-processing. In contrast, the absorption spectra of CuSCN-MTz (Figure S5c) and CuSCN-MBTz (Figure 3a) showed a clear bathochromic shift compared to the CuSCN control sample due to the MLCT transitions from Cu to the coligand states. It has been shown that the π* LUMO states of aromatic ligands are usually found at lower energies than the π* states of SCN.^17^ The MLCT absorption of CuSCN-MTz was significantly weaker in the thin film compared to the bulk, which might suggest partial loss of MTz. This is also consistent with the weak XRD signal of the CuSCN-MTz thin film.

**Figure 3:**
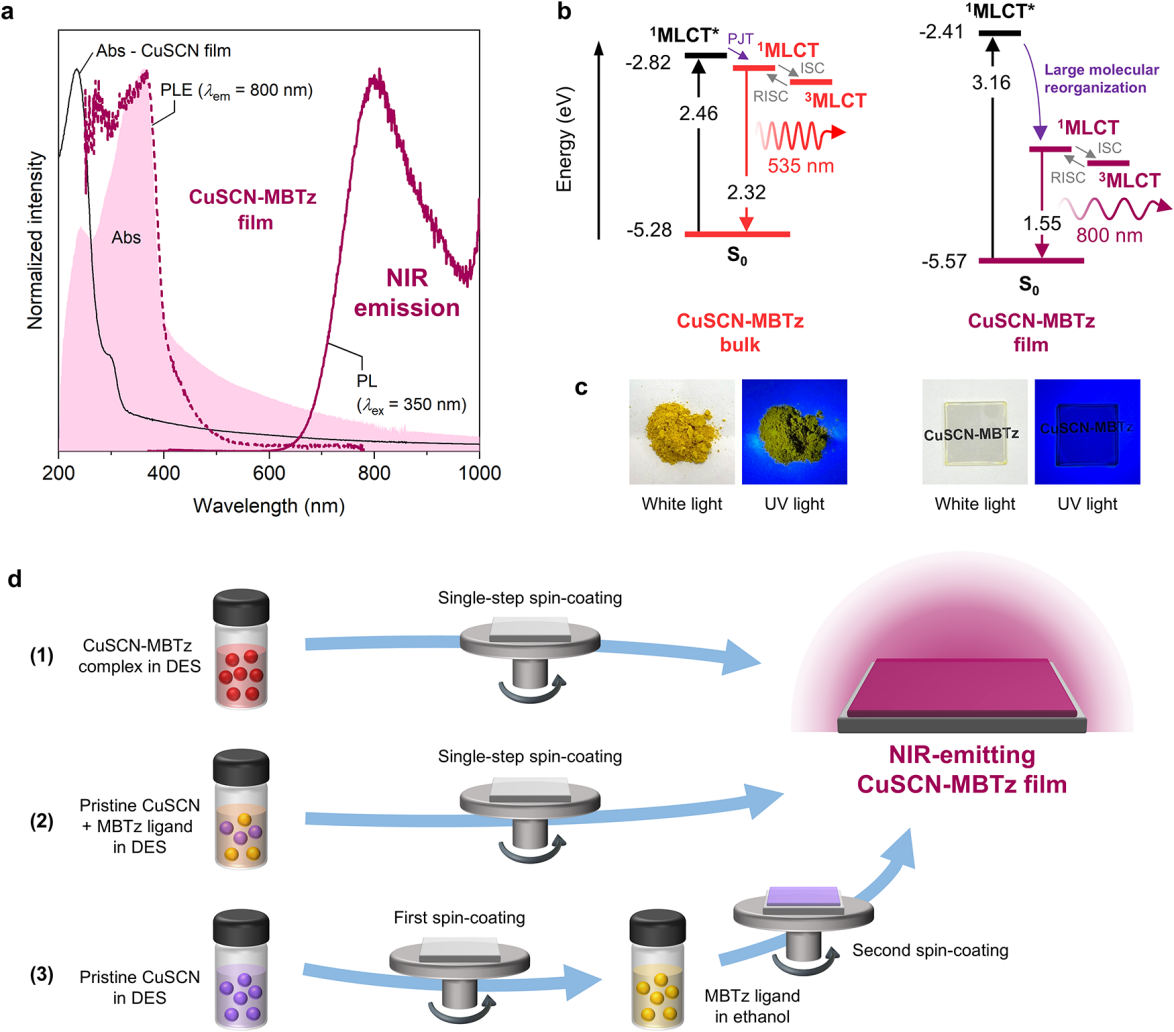
(a) Absorption, PL, and PLE spectra of thin films of CuSCN-MBTz. Absorption spectrum of a control CuSCN film is shown for reference as a solid line. (b) Energy level diagram of CuSCN-MBTz in bulk and thin-film forms. PJT: pseudo-Jahn–Teller distortion. ISC: intersystem crossing. RISC: reverse ISC. (c) Photographs of CuSCN-MBTz in bulk and thin-film forms under white light and UV light. (d) Three different routes for obtaining NIR-emitting CuSCN-MBTz film. DES: diethylsulfide, solvent used for solution-processing.

The thin films of CuSCN-MBTz are unique in this series being the only ones to show detectable emission. PL characteristics (Figure 3a) of CuSCN-MBTz thin films were strikingly different from the bulk phase; the emission peak λ_PL,max_ was drastically red-shifted from 535 to 800 nm. The PLQY was also relatively high at 22.8%. The PLE spectrum coincided well with the main peak and edge of the absorption spectrum, indicating that the emission is from the excitation of the MLCT state. TRPL results (Figure S3e) found an average lifetime of 9.3 μs. To construct the energy level diagrams of CuSCN-MBTz in both bulk and thin-film forms, the band gap was estimated from the Tauc plot (Figure S6a,b) whereas the highest occupied molecular orbital (HOMO) was obtained from the ionization potential based on photoelectron yield spectroscopy (Figure S6c,d). Combining these data with photophysical data (see next section), the resulting energy level diagrams are shown in Figure 3b. The thin films of CuSCN-MBTz notably display a rare feature, appearing transparent due to the large band gap (>3 eV) but emitting in the NIR range. The completely different energy levels and photoluminescence profile of the thin films of CuSCN-MBTz compared with the bulk (see also Figure 3c for photographs of the samples) clearly indicates a wholly distinct emission mechanism, which we elucidate further below in the next section.

Importantly, this remarkable NIR emission was consistently observed from three different film fabrication routes (Figure 3d). Route (1), as described above, proceeded by spin-coating a solution of the presynthesized CuSCN-MBTz complex. Route (2) was similar to Route (1), but the solution was instead prepared by dissolving pristine CuSCN and pristine MBTz ligand together. Finally, Route (3) involved two spin-coating steps sequentially: pristine CuSCN first, followed by pristine MBTz. Apparently, the PL and PLE spectra of the CuSCN-MBTz thin films prepared by three different routes were very similar (Figure S7a,c), indicating excellent reproducibility and photophysical stability of the emissive state. As summarized in Table 2, the peaks for the PLE and PL spectra were around ∼360–370 and ∼795–805 nm, respectively, resulting in exceptionally large excitation–emission Stokes shifts of around 15,000 cm^–1^. Compared to recent reports for copper­(I) complexes exhibiting NIR emission beyond 800 nm (Table 3),^37–39^ our CuSCN-MBTz thin films show one of the largest Stokes shifts known to date.

**Table 2:** Photophysical Properties of CuSCN-MBTz Complex in Film Form

**preparation method**	**excitation** **λ**_**PLE,max**_ **(nm)**	**emission** **λ**_**PL,max**_ **(nm)**	excitation–emission Stokes shift (cm^–^^**1**^ **)**
Route (1)	364	800	14,973
Route (2)	370	805	14,605
Route (3)	360	795	15,199
Dropcast	360	796	15,215
	430	550	5074

**Table 3:** Comparison of Photophysical Properties of Cu­(I) Complexes that Emit at λ ≥ 800 nm from Literature

**year**	**reference**	**complex**	**absorption** **λ**_**abs**_ **(nm)**	**excitation** **λ**_**PLE**_ **(nm)**	**emission** **λ**_**PL**_ **(nm)**	**PLQY Φ (%)**	**PL lifetime** **τ**_**PL**_ **(μs)**	**absorption-emission Stokes shift** (cm^–^^**1**^ **)**	**excitation–emission Stokes shift** (cm^–^^**1**^ **)**
2010	Dalton Trans. 39, 8759.	1b	662^a^	662^a^	820^a^	n/a	<0.06^a^	2911^a^	2911^a^
		3b	656^a^, 645^b^	656^a^, 645^b^	801^a^, 825^b^	n/a	<0.06^a^	2760^a^, 3383^b^	2760^a^, 3383^b^
		5b	667^a^, 661^b^	667^a^, 661^b^	822^a^, 814^b^	n/a	<0.06^a^	2827^a^, 2844^b^	2827^a^, 2844^b^
		1c	646^a^	646^a^	805^a^	n/a	<0.06^a^	3058^a^	3058^a^
2011	Inorg. Chem. 50, 11309.	C3	465^c^	460^c^	800^c^	n/a	0.005^c^	9005^c^	9239^c^
		C4	467^c^	460^c^	>800^c^	n/a	≤0.004^c^	>8913^c^	>9239^c^
2018	Polyhedron 140, 42.	C2	456^c^	456^c^	815^c^	n/a	n/a	9660^c^	9660^c^
	**this work**	CuSCN-MBTz bulk	434	473	535	n/a	5.7	4350	2450
		**CuSCN-MBTz film**	**362**	**364**	**800**	**22.8**	**9.3**	**15,124**	**14,973**

a Measured in toluene at room temperature.

b Measured in diethyl ether at room temperature.

c Measured in dichloromethane at room temperature.

### Photophysical Study of NIR Emission in CuSCN-MBTz Film

We turn our attention to why the photophysical properties of CuSCN-MBTz become so drastically different in the film state compared to the bulk state. Interestingly, thicker films prepared by drop-casting exhibited dual emissions at around 530–550 and 800 nm (Figure S8) similar to the bulk and thin-film states, respectively. The relative intensity depends strongly on the excitation wavelength. Under 350 nm excitation, the 800 nm NIR emission dominates. In contrast, under 430 nm excitation, the bulk-like emission at ∼540 nm becomes more pronounced. We note that the pristine MBTz ligand in the film state also showed two emission peaks, but at 463 and 644 nm. However, as the emissions of the drop-cast films matched well with emissions of the bulk and film states of CuSCN-MBTz, we hypothesize that there are two different forms of CuSCN-MBTz and that the thick film consists of a mixture of both.

To investigate the peculiar NIR emission from CuSCN-MBTz in more detail, we recorded the PL spectra of the complex in both bulk and thin-film forms at various excitation wavelengths. As shown in Figure 4a,b, the 2D PL mapping results are completely different between the two forms. For the bulk, the peak emission λ_PL,max_ remains at 535 nm with an excitation at 470 nm yielding the highest PL intensity. In stark contrast, λ_PL,max_ of the thin film is centered at 800 nm with the highest intensity attained from 365 nm excitation. The Stokes shift for the latter is as large as 14,897 cm^–1^, which is significantly larger than most reported metal complexes.^40–44^ Such a large Stokes shift is particularly useful for advanced optoelectronic and sensing applications,^45,46^ as it effectively minimizes self-absorption and reabsorption losses, thereby enhancing luminescence efficiency and facilitating accurate signal detection. The 2D mapping further corroborates that the emission in CuSCN-MBTz thin film originates from a different mechanism compared to the bulk. The fact that the emission peaks of both forms did not shift with the excitation source confirms that the signals are not due to scattering.

**Figure 4:**
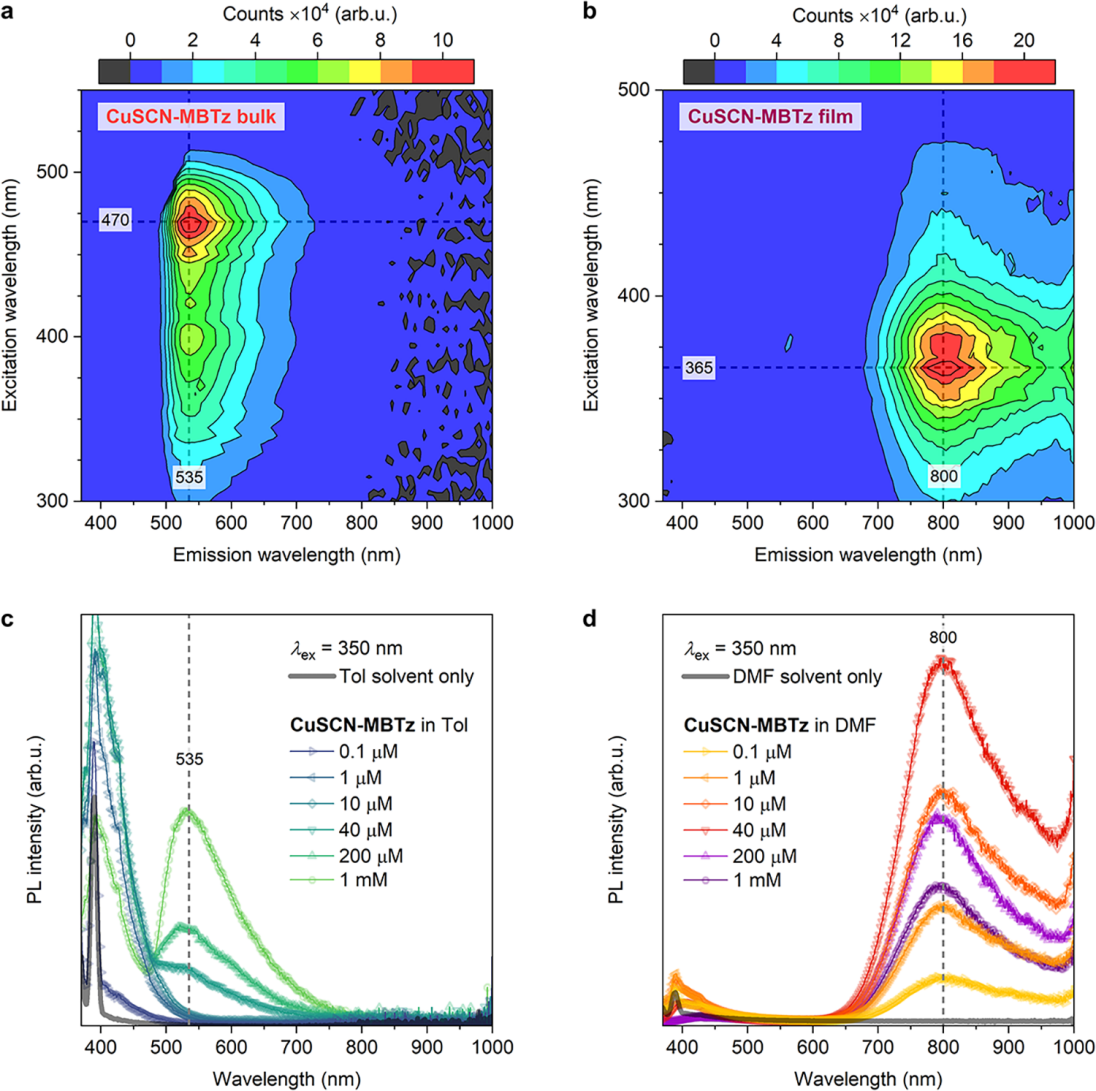
PL excitation–emission 2D mapping of CuSCN-MBTz in (a) bulk form and (b) thin-film form. PL spectra of CuSCN-MBTz at various concentrations in (c) toluene and (d) DMF.

We then performed temperature-dependent PL and TRPL for more detailed analysis (77 to 300 K). The thick film of CuSCN-MBTz prepared by drop-casting was employed in this experiment because it allowed the study of both emissive species in the same setup. Spectral data, plotted in Figure S9, showed that both peaks were blue-shifted with the increasing temperature. Peak analysis by Gaussian fitting found that the higher energy peak was shifted from ∼600 to ∼550 nm (energy increased by ∼0.17 eV) and that the lower energy peak was shifted from ∼ 855 to ∼ 830 nm (energy increased by 0.05 eV). No additional peaks were observed within the temperature range of study. Analyses of the decay kinetics, data shown in Figure S10, found that the lifetime decreased with the increasing temperature, from ∼8 to ∼6 μs for 540 nm emission and from ∼12 to ∼7 μs for 800 nm emission. Overall, the temperature dependence follows the characteristics of TADF behavior observed in Cu­(I) complexes, arising from the combination of relatively strong SOC of Cu and MLCT character.^33,47^ Theoretically, the low-temperature emission should be phosphorescence of a triplet state while the high-temperature emission should be fluorescence of a singlet state that is repopulated by reverse intersystem crossing (RISC). However, in the case of Cu­(I) complexes with MLCT character, the singlet and triplet states can be well admixed, as is the case here. Indeed, computational study (discussed below) confirms that the difference in energy between the singlet and triplet states (Δ*E*_S‑T_) is small in CuSCN-MBTz.

Furthermore, we recorded the optical properties of CuSCN-MBTz in two solvents with different polarities: toluene (nonpolar) and dimethylformamide (DMF) (polar aprotic). In the solution phase, the UV–vis absorption peak of CuSCN-MBTz was found around 325 nm in both solvents (Figure S11a,b), likely due to LC transitions which are observed at higher energies (previous section). MLCT transitions were detectable only from PLE spectra (higher signal-to-noise ratio) as discussed below. However, as shown in Figure 4c,d, the complex clearly exhibited different luminescence properties in the two solvents, also reflecting the two different solid forms. In toluene, the complex started to emit when the concentration was sufficiently high (≥40 μM), and the main peak was found at 535 nm, similar to the bulk. We can attribute this to the formation of CuSCN-MBTz microcrystals with bulk-like properties due to the limited solubility in the nonpolar solvent. In contrast, the emission shifted bathochromically to 800 nm in DMF, coinciding with the thin-film form. With higher solubility in DMF, the emissive species are likely associated with discrete molecular units. The reduction in the PL intensity at high concentrations possibly indicates the precipitation of solids. When a mixture of toluene:DMF was used as the solvent, only the 800 nm emission could be detected even with a small amount of DMF (Figure S11c). Solvatochromism was not clearly observed (Figure S11d); however, this may be due to very high affinity of CuSCN-MBTz with DMF. Our efforts of varying solvent species were not fruitful due to solubility limitations of CuSCN-MBTz in other common solvents.

From TRPL measurements (Figure S12a,b), the decay profile of the complex in toluene closely resembled that of CuSCN-MBTz in the bulk phase whereas the profile of the complex in DMF was more similar to the film state, confirming that the two emissive states are of different origins. We also compared the TRPL results of the complex in DMF excited at two different wavelengths, 350 and 470 nm. As shown in Figure S12c,d, the results of various concentrations of CuSCN-MBTz in DMF were all similar to the result of the film state (Figure S3e), and ⟨τ⟩_int_ were found to be around 8–9 μs in all cases, further corroborating that the emissive species in the thin film was analogous to the molecular state in DMF solution. As discussed in detail in the next section, the NIR emission can be explained based on molecular reorganization in the absence of crystal packing forces.

### Origin of the Large Stokes Shift and NIR Emission in CuSCN-MBTz Film

To elucidate the origin of the unusually large Stokes shift and NIR emission observed in the CuSCN–MBTz film, we performed density functional theory (DFT) calculations on the Cu_2_(SCN)_2_(MBTz)_4_ dimeric unit. The geometry of this cluster changes dramatically once it is not constrained by intermolecular forces or geometry of the crystal packing (Table S3). In the CuSCN-MBTz single crystal structure (Figure 1h), the MBTz coligands are forced face-on to each other (Figure 5a) with very weak excitonic coupling (∼0.5 meV), consistent with the absence of NIR emission in the bulk phase. In this geometry, the singlet and triplet states are close in energy at 2.58 and 2.34 eV. The values are similar to the transition energies of 2.46 eV for absorption and 2.32 eV for emission of CuSCN-MBTz bulk phase obtained from photophysical characterizations (Figure 3b). The smaller experimental values likely indicate the pseudo Jahn–Teller (PJT) effect that stabilizes the excited state.^22^ The calculated Δ*E*_S‑T_ of 0.24 eV is within the range that allows TADF.^33^ We note that these energies are from the ground state crystal geometry only; calculating for the excited state in this case would require reoptimizing the overall periodic structure, which is computationally prohibitive. However, due to the geometrical restrictions of crystal packing, the energies of the singlet and triplet states are not expected to change significantly.

**Figure 5:**
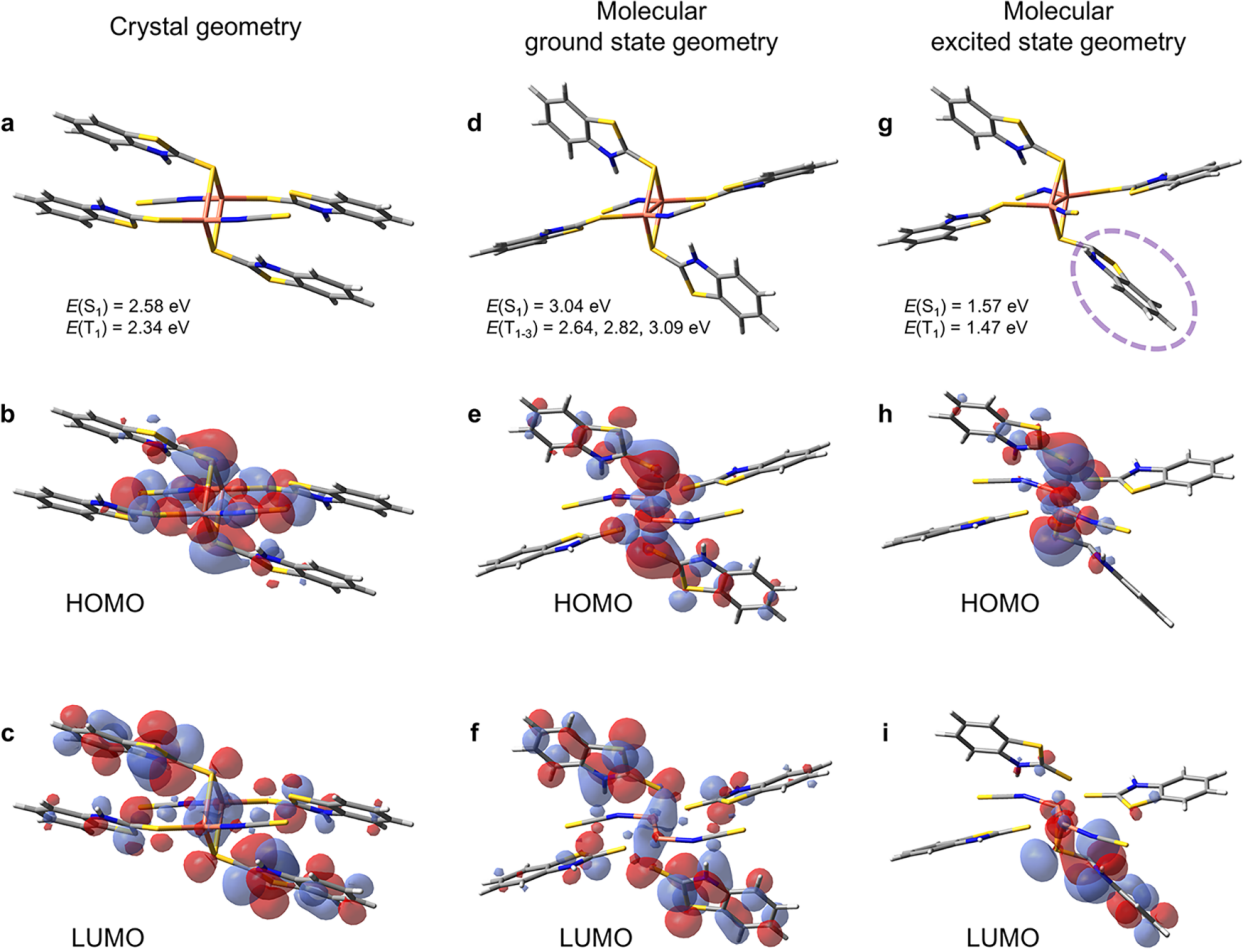
Structure and HOMO/LUMO states of the Cu_2_(SCN)_2_(MBTz)_4_ dimeric unit in (a–c) crystal geometry, (d–f) ground state S_0_ geometry without crystal packing constraint, and (g–i) excited state S_1_ geometry without crystal packing constraint, respectively. Energies of the singlet and triplet states are also included.

In contrast, when unconstrained by crystal packing, the MBTz ligands (as well as SCN ligands to some extent) become significantly twisted, pointing away from the central Cu_2_S_2_ diamond core in the S_0_ ground state geometry (Figure 5d). Moreover, the cluster reorganizes further in the S_1_ excited state geometry, with one MBTz ligand (circled in Figure 5g) showing a significant change in orientation. Crucially, the inversion symmetry is broken in the excited state. The HOMO and LUMO states that are initially distributed over the cluster (Figure 5e,f) instead become localized on the Cu_2_S_2_ core for the HOMO (Figure 5h) and one of the MBTz ligand for the LUMO (Figure 5i). Transition energies of the singlet and triplet states of the ground state geometry, which correspond to light *absorption*, were found to be 3.04 eV and between 2.64 and 3.09 eV (∼408 and ∼ 402–470 nm), respectively (Δ*E*_S‑T_ = 0.05–0.40 eV). These values agree well with the absorption spectrum of the CuSCN-MBTz film (Figure 3a). In contrast, large molecular reorganization of the excited state geometry, which corresponds to light *emission*, drastically reduces the singlet and triplet energies to 1.57 and 1.47 eV (∼790 and ∼845 nm) (Δ*E*_S‑T_ = 0.10 eV). The latter also agree well with the PL spectrum of CuSCN-MBTz in the film state and when dissolved in DMF. Relatively large S_1_-T_1_ spin–orbit coupling strengths of 16.6 meV for the ground state geometry and 5.6 meV for the excited state geometry were also found. This data together with relatively small Δ*E*_S‑T_ values support fast ISC and RISC, further corroborating the TADF characteristics as discussed earlier. Our DFT calculations strongly suggest that the observed NIR emission arises from excited-state symmetry breaking and intramolecular relaxation, processes inaccessible in the rigid crystal packing.

Additional DFT calculations in various dielectric environments (vacuum, toluene, and DMF) produced nearly identical optical properties. This also supports our analysis that ascribes the bulk-like emission observed in toluene to the formation of solid-state microcrystals. Possible excited state intramolecular proton transfer (ESIPT) pathways were also examined.^48^ Although the energy barrier was relatively small (12.2 kcal mol^–1^), the predicted emission shift was negligible, ruling out ESIPT as the NIR emission origin.

While DFT calculations establish that the large Stokes shift originates from intrinsic intramolecular relaxation, further insights can be gained from Raman spectroscopy regarding changes in the packing between the bulk and film states of CuSCN-MBTz. Figure 6 compares the experimental Raman spectra (excited at 785 nm) of CuSCN-MBTz in the bulk and film forms. Some of the Raman bands were absent from the film state, which confirm the differences in the solid-state packing between the two forms. Eigenvector analysis of the theoretical spectrum of the single crystal CuSCN-MBTz structure (Figures S13–S16 and Supporting Files S1–S2) identified dominant intermolecular N–H···S dipole–dipole (hydrogen bond-like) interactions and weaker intramolecular N–H···N contacts, as indicated in the simulated spectrum (top panel of Figure 6).

**Figure 6:**
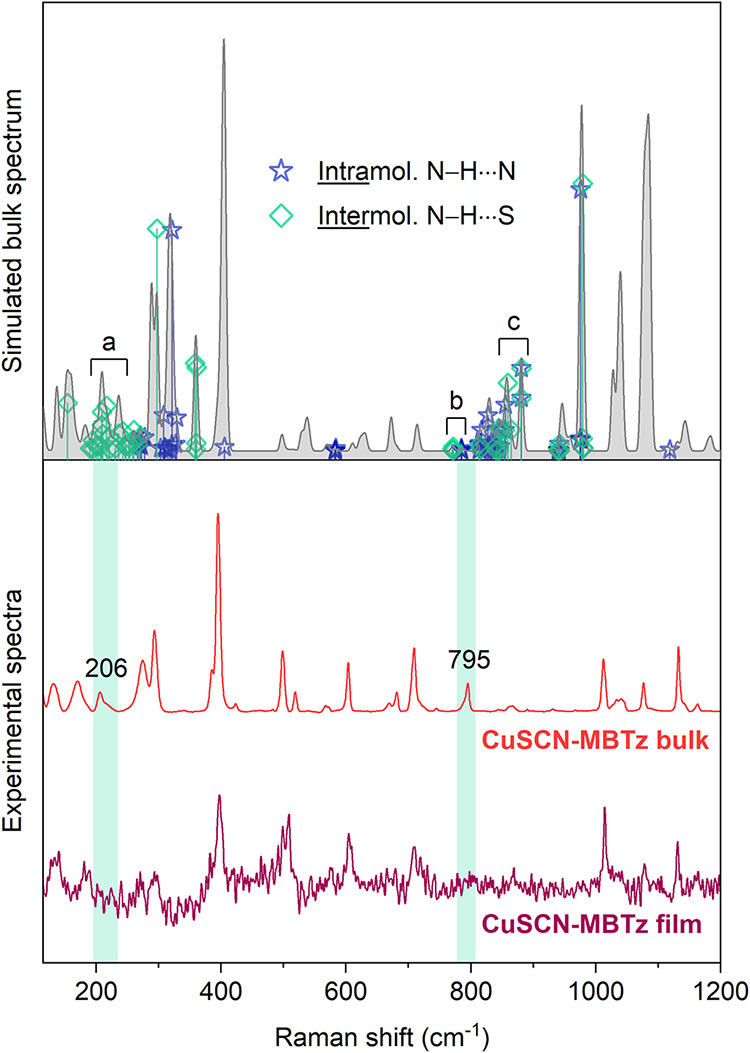
(Top) Calculated Raman spectrum of the CuSCN-MBTz single crystal structure. Vibrational modes with significant contributions are highlighted as blue stars for intramolecular N–H···N contacts and teal diamonds for intermolecular N–H···S interactions. Three regions of interest are denoted as (a), (b), and (c). (Bottom) Experimental Raman spectra of CuSCN-MBTz in the bulk and film states. The shaded areas highlight the missing bands in the film sample.

Comparing with the experimental spectra, it becomes apparent that the missing bands in the film state can be generally attributed to the intermolecular N–H···S interactions. The clearest Raman spectroscopic evidence for structural changes in CuSCN-MBTz between film and bulk appears in two features approximately at 206 and 795 cm^–1^ which are present in the bulk spectrum, but not the film (two highlighted areas in Figure 6). Based on the theoretical analysis, the feature at 206 cm^–1^ [denoted region (a) in Figure 6] arises from vibrations involving significant intermolecular N–H···S interactions with little-to-no intramolecular N–H···N contribution (Figure S16). The vibrational animations (Supporting File S2) show that these are delocalized, collective modes enabled by the intermolecular N–H···S activity. This feature therefore provides direct spectroscopic evidence for the importance of intermolecular interactions in the bulk phase.

The feature at 795 cm^–1^ in the experimental bulk spectrum is more challenging to unambiguously assign. It most likely correlates with the small theoretical peak at 770 cm^–1^ [region (b) in Figure 6]. The adjacent cluster of peaks between 800 and 900 cm^–1^ [region (c) in Figure 6] is the other possible candidate; however, based on frequency alignment, these modes are more consistent with the low broad feature at ∼850–900 cm^–1^ in the experimental spectra. Highlighted vibrations in the (b) and (c) regions arise from internal molecular modes coupled via intermolecular N–H···S interactions, imparting a mixed inter- and intramolecular character that either enhances or enables the Raman activity (animations in Supporting File S2). In the absence of this interaction, the vibrations likely become localized molecular modes with negligible Raman activity, explaining their presence in the bulk and absence in the film spectrum. A more detailed discussion of the mode analysis is available in the Supporting Information.

Taken together, the DFT and theoretical Raman analyses showed that in the loosely packed film state, reduced intermolecular coupling allows the CuSCN–MBTz dimers to reorganize upon photoexcitation. This excited-state structural relaxation breaks the inversion symmetry and stabilizes the emissive charge-transfer state, leading to the large Stokes shift and strong NIR emission which are rarely observed in other typical copper systems.^49,50^

## Conclusions

We have shown that the symmetry-breaking of the excited state can achieve low-energy photon emission, a strategy which does not require rare-earth metals or extended π-conjugation. Thin films of CuSCN-MBTz, while retaining a large band gap of >3 eV, remarkably displayed an NIR emission with a peak wavelength at 800 nm, a Stokes shift of ≈15,000 cm^–1^, and PLQY ≈ 23%. These properties were consistently reproducible via three different fabrication routes and represent one of the largest Stokes shifts and longest emission wavelengths reported for earth-abundant, Cu­(I)-based materials. Detailed experimental and theoretical investigations revealed that the NIR emission originates from molecular reorganization of the excited state which is only allowed in the thin-film form due to the absence of intermolecular, crystal-packing forces. This work demonstrates a new design guideline for tuning the optical properties of coordination materials and highlights CuSCN-based semiconductors as a promising new platform for low-cost NIR photonic and optoelectronic materials.

## Experimental and Computational Methods

### Complex Preparation

Copper­(I) thiocyanate powder (CuSCN, 99%, Sigma-Aldrich) was dissolved in diethyl sulfide (DES, 98%, Sigma-Aldrich) at a concentration of 0.05 or 0.1 M. The solution was stirred at room temperature for 12 h and then filtered through a 0.2-μm Teflon syringe filter. To prepare CuSCN-Tz and CuSCN-MTz solutions, thiazole (Tz, C_3_H_3_NS, >98%, TCI) or 2-mercaptothiazole (MTz, C_3_H_3_NS_2_, >98%, TCI) was separately added (at a concentration of 0.1 M) to the stock 0.1 M CuSCN solution to obtain CuSCN-Tz or CuSCN-MTz complex with a CuSCN:L ratio of 1:1. While for the CuSCN-BTz and CuSCN-MBTz solutions, benzothiazole (BTz, C_7_H_5_NS, >96%, TCI) or 2-mercaptobenzothiazole (MBTz, C_7_H_5_NS_2_, >98%, TCI) was separately added (at a concentration of 0.1 M) to the stock 0.05 M CuSCN solution to obtain CuSCN-BTz or CuSCN-MBTz complex with a CuSCN:L ratio of 1:2. All solutions were stirred overnight and filtered using a 0.2-μm polytetrafluoroethylene (PTFE) filter in a N_2_-filled glovebox. ***Caution!*** MBTz (CAS No. 149-30-4) can cause skin sensitization (Category 1). DES (CAS No. 352-93-2) is a volatile and flammable liquid (Category 2); can cause skin corrosion/irritation (Category 2); and can cause serious eye damage/irritation (Category 2). DES has a strong garlic-like/sulfur-based odor. All procedures involving chemicals should be carried out in a well-ventilated fume hood using appropriate personal protective equipment, in accordance with the standard chemical safety practice.

### Single-Crystal X-ray Diffraction (SCXRD)

Crystals of CuSCN-Tz, CuSCN-BTz, CuSCN-MTz, and CuSCN-MBTz were selected using a polarization microscope, immersed in Paratone Oil, then mounted on MiTeGen microloops, and analyzed at 100 K. Diffraction data were collected using a Bruker D8 Venture single-crystal diffractometer (Mo Kα, λ = 0.7107 Å). APEX3, PLATON, OLEX2, and MERCURY3 software were used for structural refinement and visualizations.

### Thermogravimetric Analysis (TGA)

TGA data were recorded using a Rigaku Thermo plus EVO2 TG-DTA 8122 under flowing N_2_ gas in the temperature range of 40 to 500 °C with a heating rate of 10 °C min^–1^. Al pans were used for all powder samples.

### Powder X-ray Diffraction (PXRD)

Manually ground samples were characterized using a Bruker D8 Advance diffractometer (Cu Kα, λ = 1.5406 Å) under ambient conditions using silicon zero background-type sample holder. The diffraction patterns were recorded for a 2θ range between 5° to 60°. Grazing incidence (GIXRD) measurements were performed at an incidence angle of 0.1°.

### Photoluminescence Spectroscopy (PL)

PL spectroscopy was conducted using an Edinburgh FLS980 spectrophotometer, with a 450-W ozone free xenon arc lamp. Photoluminescence quantum yield (PLQY) was measured using the same instrument equipped with a calibrated integrating sphere. Time-resolved photoluminescence (TRPL) was conducted using the μF2 xenon microsecond flashlamp. The emission decay profiles were fitted with a biexponential function. In this model, the PL intensity *I*(*t*) is expressed as

2 $$I\left(t\right)=B_{1}\;\exp\left(\frac{-\left(t-t_{0}\right)}{\tau_{1}}\right)+B_{2}\;\exp\left(\frac{-\left(t-t_{0}\right)}{\tau_{2}}\right)$$

where τ_1_ and τ_2_ are the lifetimes of the fast and slow decay components, respectively, and *B*_1_ and *B*_2_ are the corresponding pre-exponential factors, reflecting the relative population of the states contributing to the overall decay. The intensity-averaged PL lifetime (⟨τ⟩_int_) is calculated from the following equation:

3 $${\left\langle \tau \right\rangle }_{\mathrm{int}}=\frac{B_{1}\tau_{1}^{2}+B_{2}\tau_{2}^{2}}{B_{1}\tau_{1}+B_{2}\tau_{2}}$$

### Temperature-Dependent PL Measurements

The sample was mounted on the coldfinger of an Optistat DN-V liquid-nitrogen cryostat, allowing temperature control from 77 to 300 K. Prior to cooling with liquid nitrogen, the cryostat chamber was evacuated to provide thermal isolation and to prevent moisture condensation during low-temperature operation. The vacuum was maintained throughout the measurements, and the sample was allowed to equilibrate at each set temperature before data acquisition. The PL spectra and TRPL measurements were recorded with the same conditions as described above.

### Thin-Film Deposition

Substrates were cleaned sequentially using ultrasonication in 1%v/v detergent solution (Liquinox, Alconox Inc.), deionized water, acetone, and isopropanol, each for 30 min. Prior to use, the substrates were dried using a stream of N_2_ gas and treated with UV-ozone for wettability improvement. The solutions of CuSCN-L complexes were deposited onto the substrates by spin coating at 3000 rpm for 60 s. The resulting films were annealed at 60 °C for 30 min. The spin-coating and annealing steps were performed in a N_2_-filled glovebox.

### Ultraviolet–Visible Spectroscopy (UV–vis)

Absorption spectra of solution and film samples at room temperature were recorded using a PerkinElmer LAMBDA 1050 spectrophotometer, equipped with an integrating sphere (photomultiplier and InGaAs detectors). The optical band gaps were calculated using Tauc plots. Measurements were performed in the diffuse reflectance mode for powder samples packed into the solid sample holder. Reflectance spectra (*R*) were converted into absorbance using Kubelka–Munk function *F*(*R*):^51^

4 $$F\left(R\right)=\frac{{\left(1-R\right)}^{2}}{2R}$$

### Photoelectron Yield Spectroscopy (PYS)

The ionization potentials which correspond to the VBM or HOMO levels of solid samples were determined using a RIKEN AC-2 photoelectron spectrometer. The UV excitation was produced by a deuterium lamp with an energy range of 3.4–6.2 eV and monochromated by a grating. The total photoelectron yield was recorded as a function of excitation energy, and the ionization potentials were determined from the threshold energies of the yield^1/3^ spectra. Samples were prepared on conductive ITO substrates and measured under ambient conditions.

### Raman Spectroscopy

Raman spectra of powder and film (on fused silica) samples were recorded using a Bruker Senterra dispersive Raman microscope. The measurements were performed with a 785 nm laser excitation operated at 20 mW. All Raman spectra were obtained by a 20× objective lens and 30-s acquisition time. Data analysis was conducted using OPUS software. A blank fused silica substrate was also measured and used for background subtraction of the film sample.

### Density Functional Theory (DFT)

DFT calculations were performed in Gaussian 16 using the CAM-B3LYP^52^ exchange–correlation functional in combination with the 6–31G­(d,p) basis set. Linear response time dependent DFT (TD-DFT) calculations for excited states were performed within the Tamm-Dankoff approximation (TDA). Where included, solvent effects were included using a polarizable continuum model (integral equation formalism) with dielectric parameters for toluene or dimethylformamide. Optimized geometries were verified via inspection of the associated normal mode, which exhibited no negative frequencies. Excitonic coupling between molecules was calculated using the excitation energy transfer (EET) module in Gaussian. Spin–orbit coupling (SOC) values were calculated using the default algorithm in ORCA 5.0.3.^53,54^

### Raman Spectrum Simulation

The theoretical Raman spectrum was calculated using the finite differences method^55^ with DFT. This approach only accesses Γ-point phonons. Nuclear displacements and postprocessing were performed with the Phonopy suite (Phonopy, Phono3py, and Phonopy Spectroscopy).^56–58^ The Vienna *ab initio* Simulation Package (VASP)^59^ was used to perform single-point calculations. The PBE functional^60^ was employed with damped D3 dispersion,^61^ a planewave energy cutoff of 700 eV, and PAW–PBE pseudopotentials.^62^ This level of theory is sufficient for qualitatively accurate frequencies and intensities, but not quantitative precision. Alignment of landmark peaks was used to determine that a rigid shift of +115 cm^–1^ was most appropriate for comparisons in the 100–1500 cm^–1^ range (raw spectrum shown in Figure S13). PBE is known to systematically underpredict frequencies.^63^ However, our shift is relatively large, likely due to lacking nonanalytic macroscopic electric-field (LO-TO) corrections in our calculations, and some system specific effects.

The vibrational modes were analyzed using the vibrational eigenvectors of nuclear triplets involved in the N–H···S or N–H···N interactions. Stretching vibration intensities (δ*H*_∥_) were quantified by the displacement of the hydrogen relative to the S or N (not covalently bonded to H) projected along the axis of the interactions. The bending vibration intensities (δ*H*_⊥_) were quantified by the total displacement orthogonal to the axis. These intensities were calculated for both the intermolecular N–H···S and intramolecular N–H···N interactions. This scheme provides a useful quantitative proxy for the intensity of interactions in a given mode. Examination of the distributions of δ*H*_⊥_ and δ*H*_∥_ by mode number (Figures S14–S15) revealed three approximate regimes: the baseline (δH_∥_ <0.1, δ*H*_⊥_ <0.3); moderate activity (0.1 < δ*H*_∥_ < 0.5, 0.3 < δ*H*_⊥_ < 0.9); and high activity (δ*H*_∥_ > 0.5, *H*_⊥_ > 0.9). The vibrations meeting the high intensity threshold were classified as possessing significant interactions.

## Supplementary Material







## Notes

Additional data from SCXRD, TGA-DTA, PXRD, UV–vis, PL, TRPL, and PYS measurements; Supporting table of DFT-optimized geometries of CuSCN-MBTz; additional data from theoretical simulation of Raman spectrum and extended discussions (PDF)
Simulated Raman spectral data, vibrational mode numbers (408 modes), and calculated bond motion intensities (XLSX)
Bulk unit cell with vibrational animations of all 408 vibrational modes (ZIP)
