# Supplementary material for: Near-Infrared Luminescence from Transparent Thin Films of Copper(I) Thiocyanate Modified with 2‑Mercaptobenzothiazole via Excited-State Symmetry Breaking
Source: Inorg Chem. 2026 Apr 9;65(15):8336–49. doi: 10.1021/acs.inorgchem.5c05480 (PMC13100947; doi:10.1021/acs.inorgchem.5c05480)
Supplement: Supplementary file 1 [file ic5c05480_si_001.pdf]

Supporting Information

Near-Infrared Luminescence from Transparent Thin Films of Copper(I) Thiocyanate Modified with 2-Mercaptobenzothiazole via Excited-State Symmetry Breaking

Saran Waiprasoet,[a] Paul A. Hume,[b,c,d] James M. Scott,[e] Phattananawee Nalaoh,[a] Pongkamon Prayongkul,[a] Daniel M. Packwood,[e] Justin M. Hodgkiss,[b,d] David J. Harding,[f] and Pichaya Pattanasattayavong[a,*]

[a] Department of Materials Science and Engineering, School of Molecular Science and Engineering, Vidyasirimedhi Institute of Science and Technology (VISTEC), Rayong 21210, Thailand

[b] MacDiarmid Institute for Advanced Materials and Nanotechnology, Wellington 6012, New Zealand

[c] The Dodd-Walls Centre for Photonic and Quantum Technologies, Dunedin 9016, New Zealand

[d] School of Chemical and Physical Sciences, Victoria University of Wellington, Wellington 6012, New Zealand

[e] Institute for Integrated Cell-Material Sciences (iCeMS), Kyoto University, Kyoto 606-8317, Japan

[f] School of Chemistry, Institute of Science, Suranaree University of Technology, Nakhon Ratchasima 30000, Thailand

* Corresponding author. E-mail address: pichaya.p@vistec.ac.th

Table S1. Crystallographic data and refinement parameters of CuSCN-Tz, CuSCN-BTz, CuSCN-MTz, and CuSCN-MBTz complexes (CCDC 2487614, 2487615, 2487616, and 2487617).

Compound	**CuSCN-Tz**	**CuSCN-BTz**	**CuSCN-MTz**	**CuSCN-MBTz**
Empirical formula	$C_{12}H_9Cu_3N_6S_6$	$C_{15}H_{10}CuN_3S_3$	$C_4H_3CuN_2S_3$	$C_{30}H_{20}Cu_2N_6S_{10}$
Formula weight	620.23	391.98	238.80	912.20
Crystal system	monoclinic	orthorhombic	monoclinic	triclinic
Space group	$P2_1/c$	$P2_12_12_1$	$P2_1/c$	$P\bar{1}$
a (Å)	3.7760(4)	7.5823(3)	5.7316(4)	7.2871(4)
b (Å)	31.561(3)	12.7497(6)	19.8411(15)	14.7257(8)
c (Å)	16.3260(17)	16.1905(7)	6.7312(5)	15.7433(9)
α (°)	90	90	90	90.237(2)
β (°)	92.081(4)	90	104.882(3)	91.250(2)
γ (°)	90	90	90	92.142(2)
Volume ($Å^3$)	1944.4(4)	1565.17(12)	739.80(9)	1687.77(16)
Z	4	4	4	2
ρ_{calc} (g cm^{-3})	2.119	1.663	2.144	1.795
μ (mm^{-1})	3.907	1.792	3.711	1.914
F(000)	1224.0	792.0	472.0	920.0
Crystal size (mm^3)	0.21 × 0.18 × 0.16	0.39 × 0.32 × 0.19	0.13 × 0.06 × 0.02	0.16 × 0.08 × 0.03
Radiation	Mo Kα (λ = 0.71073 Å)			
Temperature (K)	100.00			
2θ range for data collection (°)	4.606 to 56.562	4.066 to 60.064	4.106 to 53.49	5.176 to 56.564
Index ranges	-5 ≤ h ≤ 5, -42 ≤ k ≤ 42, -20 ≤ l ≤ 21	-10 ≤ h ≤ 8, -17 ≤ k ≤ 17, -22 ≤ l ≤ 22	-7 ≤ h ≤ 7, -24 ≤ k ≤ 25, -7 ≤ l ≤ 8	-9 ≤ h ≤ 9, -19 ≤ k ≤ 19, -20 ≤ l ≤ 20
Reflections collected	38006	22498	10381	55962
Independent reflections	4780 [R_{int} = 0.0451, R_{sigma} = 0.0251]	4493 [R_{int} = 0.0348, R_{sigma} = 0.0296]	1574 [R_{int} = 0.0421, R_{sigma} = 0.0282]	8384 [R_{int} = 0.0553, R_{sigma} = 0.0391]
Data/restraints/ parameters	4780/39/255	4493/0/200	1574/0/91	8384/0/458
Goodness-of-fit on F^2	1.265	1.050	1.118	1.020
Final R indexes [I>=2σ (I)]	R_1 = 0.0349, wR_2 = 0.0826	R_1 = 0.0182, wR_2 = 0.0448	R_1 = 0.0295, wR_2 = 0.0617	R_1 = 0.0286, wR_2 = 0.0550
Final R indexes [all data]	R_1 = 0.0371, wR_2 = 0.0834	R_1 = 0.0194, wR_2 = 0.0452	R_1 = 0.0371, wR_2 = 0.0639	R_1 = 0.0462, wR_2 = 0.0596
Largest diff. peak/hole (e $Å^{-3}$)	0.86/-0.51	0.25/-0.31	0.56/-0.51	0.45/-0.35

Table S2. Fitting parameters for the time-resolved photoluminescence data. The emission was fitted with the biexponential decay function: $I(t) = B_1\exp[-(t-t_0)/\tau_1] + B_2\exp[-(t-t_0)/\tau_2]$, where I is the intensity, t is the time, t_0 is the starting time of the decay, B_1 and B_2 are the amplitudes (percentages in the table represent the relative contributions), and τ_1 and τ_2 are the lifetimes. The intensity average lifetime $\langle\tau\rangle_{\mathrm{int}}$ was calculated from: $\langle\tau\rangle_{\mathrm{int}} = (B_1\tau_1^2 + B_2\tau_2^2)/(B_1\tau_1 + B_2\tau_2)$.

Sample	B_1 (arb.u.)	τ_1 (μs)	B_2 (arb.u.)	τ_2 (μs)	$\langle\tau\rangle_{\mathrm{int}}$ (μs)
**CuSCN-Tz bulk**	560.22 (82%)	1.86	119.14 (18%)	13.74	9.12
**CuSCN-MTz bulk**	621.04 (93%)	3.03	46.05 (7%)	14.50	6.03
**CuSCN-BTz bulk**	570.88 (95%)	1.49	27.83 (5%)	11.76	4.35
**CuSCN-MBTz bulk**	523.99 (95%)	1.89	25.12 (5%)	15.51	5.74
**CuSCN-MBTz film**	594.30 (74%)	4.16	211.31 (26%)	13.67	9.28

Table S3. DFT-optimized geometries of CuSCN-MBTz.

**Ground state – Vacuum**							
Cu	0.05257900	0.44117700	-1.11596900	Cu	-0.05282000	-0.44125500	1.11632300
S	-0.09057800	1.94056500	0.66643700	S	0.09039900	-1.94068500	-0.66634100
S	-3.87366300	-0.24525600	-3.59619700	S	3.87310300	0.24619700	3.59676500
S	1.89038100	1.04352700	-2.44187000	S	-1.89036300	-1.04402300	2.44195900
S	3.48342500	1.42519100	0.12896700	S	-3.48377200	-1.42449100	-0.12881500
S	-2.20900700	3.87063000	1.61292600	S	2.20905000	-3.87044600	-1.61291100
N	-1.49940600	0.36407700	-2.22853100	N	1.49928000	-0.36401400	2.22870500
N	4.28406700	2.19122300	-2.15842700	N	-4.28365200	-2.19249000	2.15817600
H	4.28438300	2.36141600	-3.15619900	H	-4.28365700	-2.36348000	3.15581100
N	-2.58825800	2.26959900	-0.32157200	N	2.58831000	-2.26905500	0.32129500
H	-2.37265600	1.57834600	-1.05331200	H	2.37261500	-1.57791200	1.05312300
C	-2.47202900	0.09695600	-2.83838000	C	2.47172300	-0.09655900	2.83868000
C	3.22428300	1.57718100	-1.58927900	C	-3.22423300	-1.57761500	1.58925900
C	5.05016500	2.21041600	0.01114200	C	-5.05012700	-2.21052100	-0.01126700
C	5.32385500	2.56311900	-1.31121700	C	-5.32342500	-2.56427300	1.31089100
C	6.50895400	3.20238600	-1.65619900	C	-6.50817800	-3.20432500	1.65561300
H	6.71708000	3.47531400	-2.68564100	H	-6.71604000	-3.47807500	2.68489000
C	7.41332000	3.47881400	-0.64067100	C	-7.41256600	-3.48047100	0.64003300
H	8.34568100	3.97785400	-0.88297400	H	-8.34465800	-3.98010600	0.88214500
C	7.14269100	3.12553300	0.68402300	C	-7.14231200	-3.12616600	-0.68446900
H	7.86764800	3.35261200	1.45827900	H	-7.86729000	-3.35308000	-1.45875600
C	5.95772800	2.48708000	1.02529000	C	-5.95770200	-2.48692700	-1.02547700
H	5.73409300	2.20240900	2.04794000	H	-5.73432400	-2.20149000	-2.04798100
C	-1.63755200	2.60431800	0.55297400	C	1.63754500	-2.60406000	-0.55309900
C	-3.78704900	2.96589100	-0.23645000	C	3.78713900	-2.96532200	0.23623600
C	-4.90723300	2.79493700	-1.04385200	C	4.90737200	-2.79417700	1.04352900
H	-4.90674500	2.05447000	-1.83911400	H	4.90688300	-2.05358300	1.83868100
C	-6.00563100	3.60218500	-0.79134700	C	6.00579900	-3.60140100	0.79108200
H	-6.89374800	3.49415000	-1.40482700	H	6.89395300	-3.49322100	1.40448600
C	-5.98831600	4.55103300	0.23618000	C	5.98847900	-4.55040000	-0.23630400
H	-6.86291300	5.16879800	0.41054200	H	6.86310700	-5.16813300	-0.41063100
C	-4.86735000	4.71657100	1.03771300	C	4.86747300	-4.71612300	-1.03774100
H	-4.85443100	5.45326100	1.83371000	H	4.85454500	-5.45293500	-1.83362400
C	-3.76156200	3.91163500	0.79048600	C	3.76164700	-3.91122600	-0.79054800
**Ground state – Toluene**							
Cu	0.06762300	0.42509100	-1.12094100	Cu	-0.06761600	-0.42530100	1.12085200
S	-0.09609500	1.95452700	0.62518200	S	0.09607000	-1.95475500	-0.62520800
S	-3.76238100	-0.33820300	-3.73262400	S	3.76248100	0.33853700	3.73222300
S	1.92903300	0.96695300	-2.43976400	S	-1.92891000	-0.96732300	2.43983400
S	3.49711300	1.45084700	0.12915700	S	-3.49705800	-1.45060100	-0.12912800
S	-2.23490700	3.87086900	1.55782600	S	2.23507600	-3.87090900	-1.55783100
N	-1.46334700	0.33133400	-2.26306900	N	1.46333500	-0.33149100	2.26307000
N	4.34158500	2.08880800	-2.17914100	N	-4.34162000	-2.08885300	2.17906200
H	4.36639300	2.21228500	-3.18443000	H	-4.36646700	-2.21248400	3.18432800
N	-2.60057400	2.24521600	-0.35697700	N	2.60040400	-2.24549100	0.35724000
H	-2.38105000	1.54846800	-1.07968500	H	2.38077000	-1.54876200	1.07995100
C	-2.40609600	0.04200800	-2.90677500	C	2.40611700	-0.04202200	2.90666500
C	3.26717900	1.52288700	-1.59793800	C	-3.26716600	-1.52292100	1.59796900
C	5.07847700	2.20364700	-0.00120600	C	-5.07850000	-2.20327500	0.00109300
C	5.37543800	2.48500400	-1.33524200	C	-5.37549500	-2.48482000	1.33508100
C	6.57546000	3.08664200	-1.69663500	C	-6.57556300	-3.08642700	1.69637200
H	6.79860100	3.30346500	-2.73576600	H	-6.79871400	-3.30340900	2.73546800
C	7.47101500	3.39839500	-0.68403800	C	-7.47113400	-3.39795400	0.68371800
H	8.41477800	3.86906900	-0.93789600	H	-8.41493300	-3.86860400	0.93748900
C	7.17757400	3.11640500	0.65367200	C	-7.17765900	-3.11577500	-0.65394500
H	7.89646300	3.37007600	1.42516500	H	-7.89655400	-3.36928200	-1.42548500
C	5.97779800	2.51579300	1.01063900	C	-5.97783400	-2.51519900	-1.01080800
H	5.74027200	2.28938100	2.04442700	H	-5.74028800	-2.28865600	-2.04456500
C	-1.65363000	2.60212900	0.51131100	C	1.65360700	-2.60232100	-0.51124900
C	-3.80727500	2.92994000	-0.27755700	C	3.80715100	-2.93014400	0.27789100
C	-4.92725300	2.73982400	-1.08079800	C	4.92702000	-2.74005200	1.08129000

H	-4.92139800	1.99365000	-1.86980100	H	4.92104200	-1.99392600	1.87033600
C	-6.03370700	3.53784700	-0.83466300	C	6.03354500	-3.53799100	0.83520400
H	-6.92181600	3.41492200	-1.44508500	H	6.92157700	-3.41508600	1.44574200
C	-6.02358300	4.49733800	0.18369600	C	6.02359300	-4.49737200	-0.18326100
H	-6.90402800	5.10770200	0.35347200	H	6.90408800	-5.10767700	-0.35298900
C	-4.90294900	4.68320600	0.98118900	C	4.90307300	-4.68320100	-0.98092500
H	-4.89572100	5.42812400	1.76921800	H	4.89598700	-5.42802300	-1.76904700
C	-3.78969500	3.88649700	0.73911300	C	3.78974800	-3.88657400	-0.73890000

Ground state – DMF

Cu	0.09470700	0.34937800	-1.14468000	Cu	-0.09462800	-0.34884800	1.14471300
S	-0.10886900	1.99980900	0.46315700	S	0.10851800	-1.99980000	-0.46295700
S	-3.48886800	-0.74170300	-3.97004200	S	3.48892400	0.74116100	3.97055600
S	2.00064100	0.74544200	-2.45099100	S	-2.00050900	-0.74572000	2.45123900
S	3.50445800	1.50576500	0.09181300	S	-3.50430700	-1.50562900	-0.09165600
S	-2.28040500	3.91393800	1.31686700	S	2.28011200	-3.91375200	-1.31708600
N	-1.39645800	0.17582400	-2.32925800	N	1.39662700	-0.17553300	2.32916600
N	4.45028100	1.80384400	-2.24270800	N	-4.45006200	-1.80433800	2.24281200
H	4.52120600	1.79270000	-3.25444100	H	-4.52098600	-1.79341800	3.25454700
N	-2.63641500	2.17292000	-0.49389900	N	2.63609800	-2.17295700	0.49389400
H	-2.41632100	1.43727500	-1.17358200	H	2.41604700	-1.43732500	1.17357200
C	-2.25766500	-0.21098000	-3.03115200	C	2.25778100	0.21091200	3.03131800
C	3.33963600	1.35928000	-1.63731800	C	-3.33947300	-1.35952300	1.63750500
C	5.11740400	2.18073200	-0.07094300	C	-5.11718000	-2.18078800	0.07096700
C	5.47022500	2.27151100	-1.41770200	C	-5.46997100	-2.27191800	1.41771000
C	6.70562600	2.77580600	-1.80937400	C	-6.70531400	-2.77643900	1.80927100
H	6.97259200	2.84307700	-2.85803400	H	-6.97226300	-2.84399400	2.85791800
C	7.57787800	3.18641800	-0.81235700	C	-7.57753100	-3.18690600	0.81216100
H	8.54824800	3.58410600	-1.08881700	H	-8.54785500	-3.58476600	1.08853700
C	7.22863400	3.09572400	0.53941100	C	-7.22831500	-3.09585600	-0.53958900
H	7.93147000	3.42307900	1.29766200	H	-7.93113000	-3.42310600	-1.29790500
C	5.99444400	2.59168300	0.92594200	C	-5.99418000	-2.59159700	-0.92601300
H	5.71871700	2.51567400	1.97206200	H	-5.71843700	-2.51530200	-1.97211200
C	-1.68813200	2.60461400	0.33600300	C	1.68778200	-2.60459900	-0.33602300
C	-3.86172500	2.82891100	-0.42810100	C	3.86144500	-2.82884300	0.42793800
C	-4.99474600	2.56536100	-1.19170800	C	4.99449700	-2.56529100	1.19149900
H	-4.99174000	1.77739200	-1.93729400	H	4.99146700	-1.77740800	1.93717700
C	-6.11733300	3.34591200	-0.96412800	C	6.11713300	-3.34572600	0.96374900
H	-7.01566800	3.16433900	-1.54388500	H	7.01548900	-3.16415000	1.54347100
C	-6.11090900	4.36210500	-0.00162000	C	6.11072600	-4.36180300	0.00111900
H	-7.00411600	4.95678600	0.15523000	H	7.00396900	-4.95639600	-0.15585800
C	-4.97857400	4.62268100	0.75691400	C	4.97836100	-4.62237600	-0.75737600
H	-4.97336900	5.41013500	1.50193400	H	4.97316600	-5.40973500	-1.50249700
C	-3.85006500	3.84202000	0.53220700	C	3.84980700	-3.84183000	-0.53249700

Excited state – Vacuum

Cu	0.02716300	0.23992000	-1.14595700	Cu	0.00726600	-0.43471700	1.12393000
S	-0.27263500	1.70648500	0.49752200	S	0.17414600	-2.11720300	-0.50890800
S	-3.73428700	-0.46440400	-3.79103000	S	3.87420200	0.23218100	3.71976900
S	2.00271500	0.83249300	-2.40177600	S	-1.76510700	-1.24375500	2.52869800
S	3.42136700	1.43185000	0.23582300	S	-3.42603900	-1.26366000	-0.03759600
S	-2.85469100	2.55294900	1.89802100	S	2.47376900	-3.63150600	-1.77073200
N	-1.39079700	0.22632000	-2.41737600	N	1.57878200	-0.51677100	2.29445000
N	4.19835900	2.29893500	-2.01936300	N	-4.22943100	-2.19613500	2.18287900
H	4.20902200	2.47598000	-3.01623800	H	-4.22473600	-2.45439600	3.16159800
N	-2.63350500	2.41807600	-0.66768500	N	2.72435200	-2.28648300	0.36380200
H	-2.38819900	2.00299000	-1.56073600	H	2.46098100	-1.68345200	1.16176300
C	-2.36031200	-0.07696200	-3.01363100	C	2.51605100	-0.18825000	2.93234900
C	3.21292100	1.54746100	-1.48941700	C	-3.14875100	-1.59193200	1.64929500
C	4.86654000	2.42660700	0.17825900	C	-5.01903700	-1.99934700	0.03227000
C	5.14202700	2.81305500	-1.13377000	C	-5.29653200	-2.44562700	1.32455900
C	6.23919700	3.61154700	-1.43555000	C	-6.50684700	-3.05555300	1.63376500
H	6.44845200	3.91114300	-2.45721800	H	-6.71776700	-3.40058200	2.64072700
C	7.05312300	4.01192700	-0.38604600	C	-7.43193900	-3.20652500	0.61120800
H	7.91529600	4.63692300	-0.59301700	H	-8.38477200	-3.67948300	0.82434100
C	6.78043700	3.62500000	0.92936400	C	-7.15666700	-2.76011500	-0.68468400

H	7.43416200	3.95245800	1.73050500	H	-7.89838300	-2.89109100	-1.46521400
C	5.68373900	2.82802700	1.22743900	C	-5.94775700	-2.15014300	-0.98988800
H	5.46101800	2.51716700	2.24261000	H	-5.71831600	-1.79699200	-1.98989900
C	-2.02561400	1.91726200	0.47260600	C	1.80187400	-2.60836800	-0.53548200
C	-3.80458200	3.11634200	-0.46926900	C	3.99743600	-2.80258500	0.15601400
C	-4.67098800	3.59957500	-1.44715300	C	5.11906300	-2.59732300	0.95429800
H	-4.46502800	3.42266800	-2.49751900	H	5.05962600	-1.97408000	1.84227000
C	-5.80184300	4.29692700	-1.04117000	C	6.29774600	-3.21143900	0.56397800
H	-6.48577800	4.67754600	-1.79291700	H	7.19074500	-3.07321000	1.16386800
C	-6.07224300	4.51114600	0.30947800	C	6.35678100	-4.00507700	-0.58787000
H	-6.96039900	5.05980800	0.60426900	H	7.29441700	-4.47377500	-0.86692000
C	-5.21204300	4.01713400	1.28704400	C	5.23633500	-4.20277800	-1.38085500
H	-5.42302600	4.17194400	2.34017500	H	5.28493700	-4.81631700	-2.27377300
C	-4.08336100	3.31822400	0.88961800	C	4.04857100	-3.58998100	-0.99567300
**Excited state – Toluene**							
Cu	0.04338900	0.21976900	-1.12444600	Cu	-0.02998100	-0.43146600	1.14910100
S	-0.27830600	1.70431300	0.50155500	S	0.14799500	-2.12050100	-0.47015400
S	-3.62196400	-0.52072100	-3.91480400	S	3.73843400	0.31268200	3.86869000
S	2.02633200	0.76673900	-2.37571200	S	-1.80841300	-1.20806300	2.55096600
S	3.42569900	1.45580900	0.25044600	S	-3.45993400	-1.23310800	-0.01951300
S	-2.88533800	2.53391000	1.86120300	S	2.44911400	-3.62131400	-1.74266500
N	-1.36280600	0.22044800	-2.42408700	N	1.53283600	-0.49587600	2.33488200
N	4.26644400	2.17924300	-2.02992000	N	-4.26583000	-2.18628100	2.18899700
H	4.30490300	2.30598600	-3.03478200	H	-4.27116100	-2.45448900	3.16606500
N	-2.60505500	2.44948000	-0.70466700	N	2.70923000	-2.27068400	0.38489200
H	-2.35749000	2.04032200	-1.59919500	H	2.45068300	-1.67139100	1.18430700
C	-2.29672100	-0.10037500	-3.06348000	C	2.43523000	-0.14581700	3.00899400
C	3.25183900	1.49084800	-1.48093500	C	-3.19181600	-1.56987700	1.66631500
C	4.90344000	2.39949800	0.17062000	C	-5.04434900	-1.98705700	0.03347800
C	5.21245100	2.70760100	-1.15444600	C	-5.32466700	-2.44212500	1.32180900
C	6.34088300	3.45279900	-1.47851500	C	-6.52991900	-3.06712000	1.62187400
H	6.57560400	3.69145000	-2.51029600	H	-6.74100100	-3.41880400	2.62604300
C	7.15070900	3.87967300	-0.43673300	C	-7.44655400	-3.22364300	0.59299200
H	8.03676100	4.46402100	-0.66015400	H	-8.39521300	-3.70811600	0.79783900
C	6.84461200	3.57094800	0.89271700	C	-7.16857900	-2.76879300	-0.70029000
H	7.49657900	3.91772100	1.68698400	H	-7.90394100	-2.90556200	-1.48568900
C	5.71725100	2.82748100	1.21249200	C	-5.96501100	-2.14419900	-0.99566200
H	5.47151800	2.57990900	2.23950600	H	-5.73673800	-1.78752000	-1.99451600
C	-2.02703600	1.91736400	0.44001500	C	1.78511900	-2.60226100	-0.50680800
C	-3.76913700	3.16563600	-0.51580600	C	3.98483000	-2.77969100	0.16771800
C	-4.60111900	3.69026800	-1.50186600	C	5.11350300	-2.56759600	0.95446100
H	-4.37031800	3.54019500	-2.55124200	H	5.06157000	-1.94743500	1.84410900
C	-5.72917200	4.40006400	-1.10712900	C	6.29177200	-3.17469900	0.55309700
H	-6.38594900	4.81267400	-1.86614400	H	7.18990100	-3.03103400	1.14367000
C	-6.02879700	4.58737900	0.24129000	C	6.34368900	-3.96943100	-0.59915800
H	-6.91336700	5.14593000	0.52802800	H	7.28129300	-4.43246300	-0.88691700
C	-5.20108500	4.05483100	1.22725800	C	5.21710300	-4.17547700	-1.38066800
H	-5.43401600	4.19003000	2.27839200	H	5.25956400	-4.78986200	-2.27306100
C	-4.07667900	3.34349700	0.83964500	C	4.02993200	-3.56849100	-0.98308000
**Excited state – DMF**							
Cu	0.11822900	0.50927200	-1.11925400	Cu	-0.04921300	-0.33518200	1.06300000
S	-0.07717700	2.02688600	0.64459200	S	0.38057700	-1.67371100	-0.66338800
S	-3.14906300	0.20696100	-4.49138500	S	2.33163000	-0.77321900	5.08006700
S	1.88471100	1.39181400	-2.45524700	S	-2.18363000	-0.85083300	2.15667100
S	3.60519700	1.23505200	0.07150800	S	-3.37825800	-1.55540700	-0.56657900
S	-2.39121100	3.34901400	2.07483100	S	3.09441800	-2.24160400	-1.94978700
N	-1.39911400	0.71702700	-2.35468200	N	1.12896900	-0.65382400	2.54394100
N	4.31088000	2.43046500	-2.04939100	N	-4.54399600	-1.99700600	1.63917300
H	4.28260600	2.79841600	-2.99435000	H	-4.72104800	-2.03329100	2.63740500
N	-2.65829400	2.20175700	-0.16636700	N	2.66548000	-2.45516500	0.59116100
H	-2.40320800	1.69226800	-1.02511800	H	2.35058900	-2.16335300	1.51022900
C	-2.11982700	0.50143100	-3.26146400	C	1.61988400	-0.69921700	3.61100700
C	3.27997400	1.72535400	-1.56698600	C	-3.39975300	-1.48020200	1.17343100
C	5.15892200	2.05451100	0.05656400	C	-4.96372500	-2.30823700	-0.60327200

C	5.38376000	2.64311000	-1.18781500	C	-5.44976200	-2.47219400	0.69381300
C	6.55500000	3.34269100	-1.45945200	C	-6.69375100	-3.04561000	0.93441100
H	6.72042400	3.79600800	-2.43043100	H	-7.06400400	-3.16877500	1.94603700
C	7.49587400	3.43673100	-0.44573200	C	-7.43719900	-3.45036900	-0.16371200
H	8.41861700	3.97664800	-0.62765700	H	-8.41100000	-3.90108200	-0.00690600
C	7.27539400	2.84820700	0.80471100	C	-6.95410800	-3.28689600	-1.46682300
H	8.02883500	2.93816500	1.57946200	H	-7.55794000	-3.61193700	-2.30687800
C	6.10635900	2.14961400	1.07010500	C	-5.71234100	-2.71394300	-1.70220800
H	5.93557700	1.69265800	2.03821600	H	-5.33649500	-2.58374500	-2.71080300
C	-1.73293500	2.47110800	0.74106500	C	2.12996500	-1.81556300	-0.52049900
C	-3.94226000	2.65827600	0.11880400	C	3.86735200	-3.10767000	0.39340800
C	-5.08252200	2.50143100	-0.66498900	C	4.65817400	-3.71870600	1.36320300
H	-5.03955400	1.97912300	-1.61433200	H	4.35756000	-3.70455900	2.40554700
C	-6.26478200	3.03824000	-0.18484300	C	5.83496200	-4.34262400	0.96215700
H	-7.17110700	2.93367800	-0.77074800	H	6.46014700	-4.82135700	1.70883900
C	-6.31101800	3.71348900	1.04230600	C	6.22095700	-4.36157700	-0.37718000
H	-7.25251100	4.12329400	1.39086900	H	7.14189300	-4.85418700	-0.66982200
C	-5.17451800	3.86812300	1.82010300	C	5.43161900	-3.74549300	-1.34678800
H	-5.21132200	4.39052900	2.76900400	H	5.73071600	-3.75126200	-2.38967000
C	-3.98328100	3.32982700	1.34196400	C	4.25978500	-3.12015700	-0.95111000

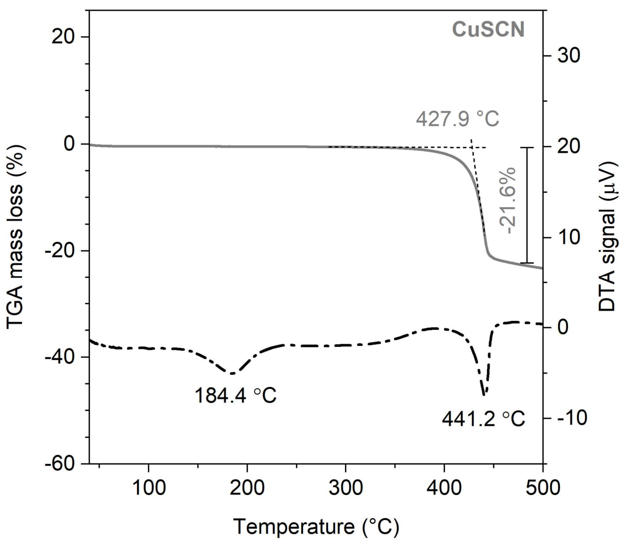

Figure S1. TGA-DTA results of reference CuSCN. The observed mass loss of 21.6% is close to the combined mass fractions of C and N (calc. 21.4%), suggesting the release of cyanogen gas $(CN)_2$ as the main decomposition product.[1] We note that partial loss of sulfur is also a possibility as reported in literature.[2] However, the remaining solids after the decomposition is likely a form of copper sulfide, which can exist in several phases at temperatures higher than 500 °C.[3]

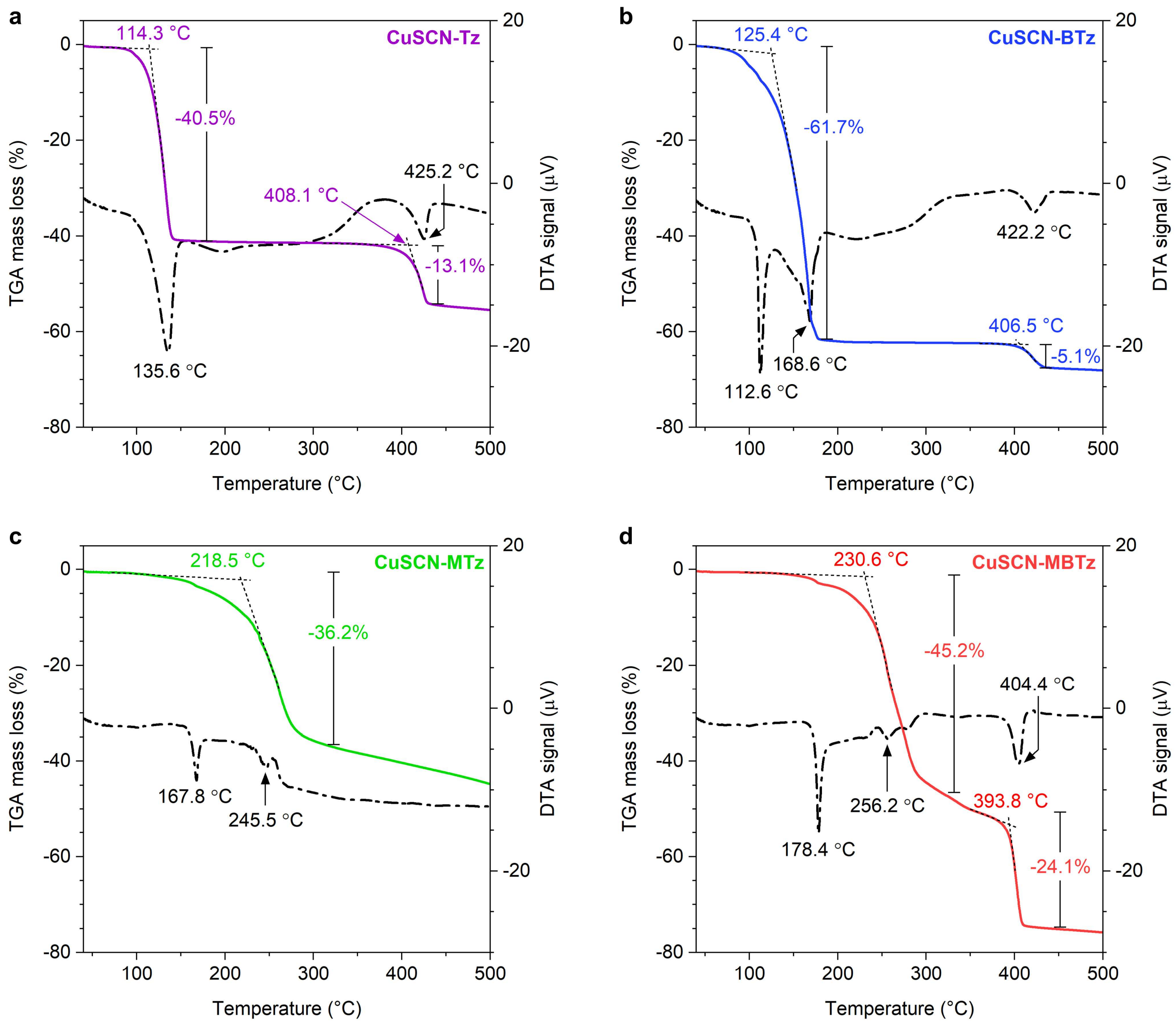

Figure S2. Thermogravimetric analysis (TGA) and differential thermal analysis (DTA) results of (a) CuSCN-Tz, (b) CuSCN-BTz, (c) CuSCN-MTz and (d) CuSCN-MBTz bulk powder samples under nitrogen atmosphere. Reported decomposition temperatures in the main text are rounded to the nearest 5 °C.

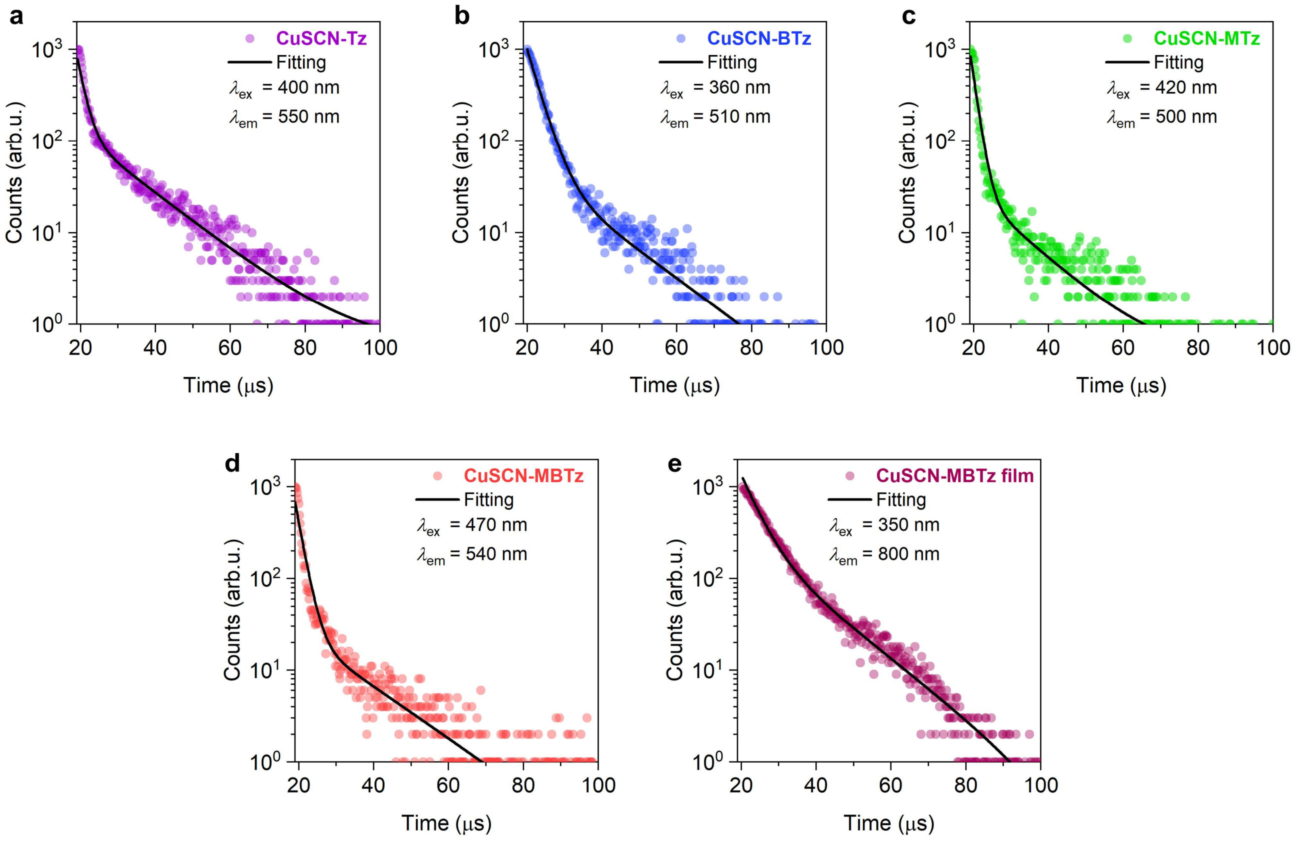

Figure S3. Time-resolved photoluminescence (TRPL) spectra (scattered points) of (a) CuSCN-Tz, (b) CuSCN-BTz, (c) CuSCN-MTz, and (d) CuSCN-MBTz in bulk powder form. (e) TRPL spectrum of CuSCN-MBTz in thin-film form. Wavelengths of the excitation source (λ_{ex}) and the monitored emission (λ_{em}) are annotated in the plots. Data were fitted with a biexponential decay as shown in black solid lines.

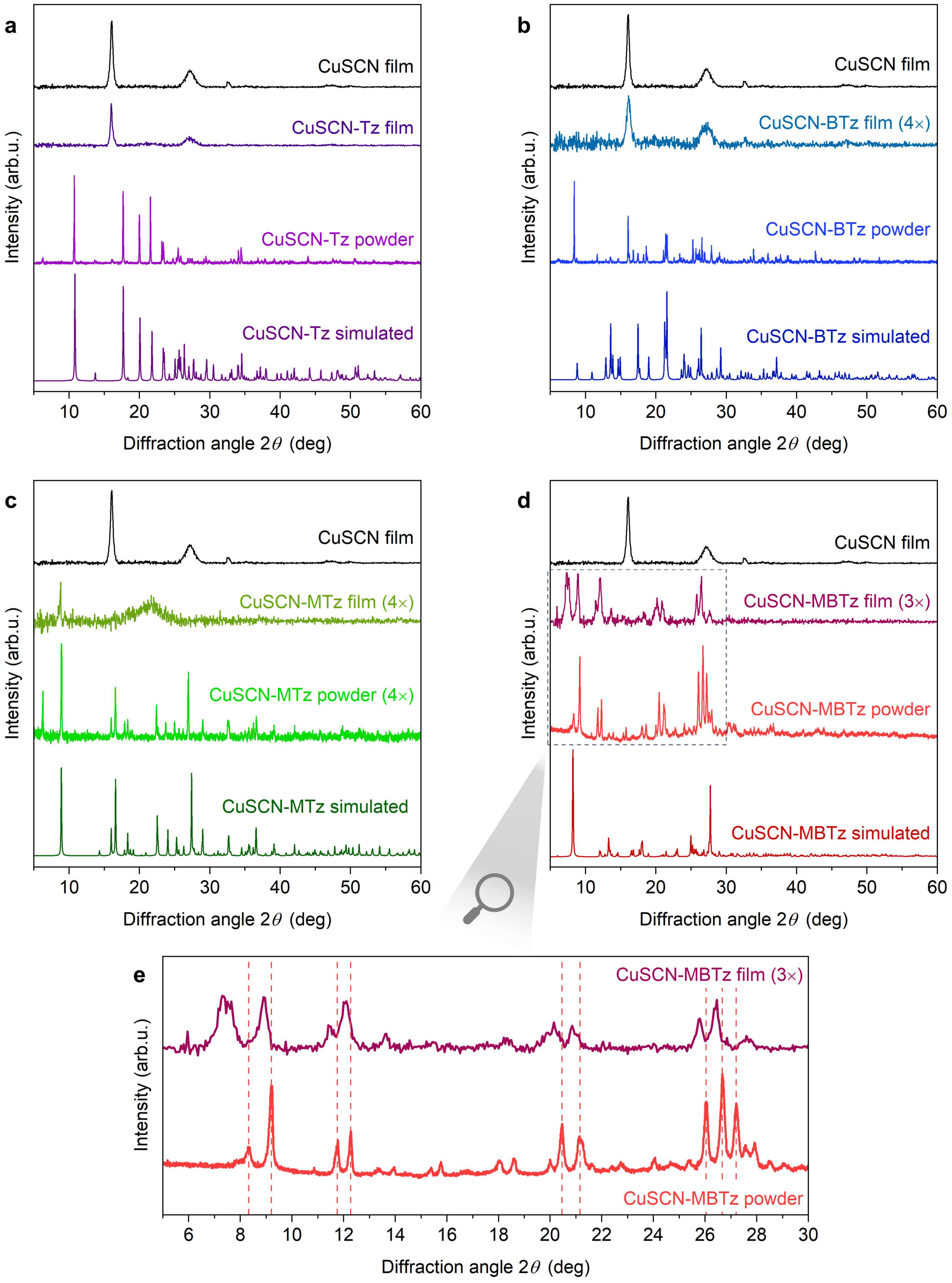

Figure S4. X-ray diffractograms of (a) CuSCN-Tz, (b) CuSCN-BTz, (c) CuSCN-MTz, and (d) CuSCN-MBTz complexes in thin-film and powder forms. Data of CuSCN film also processed with the same procedure and simulated XRD patterns of the single crystal structures are shown for reference. Multiplication factors were applied to some of the data (as annotated) to enhance visibility. (e) Close-up comparison of CuSCN-MBTz between thin-film and powder forms in the 2θ range of 5° to 30° [dashed box in panel (d)]. The vertical dashed lines mark some high-intensity peaks of CuSCN-MBTz powder sample to guide the eye.

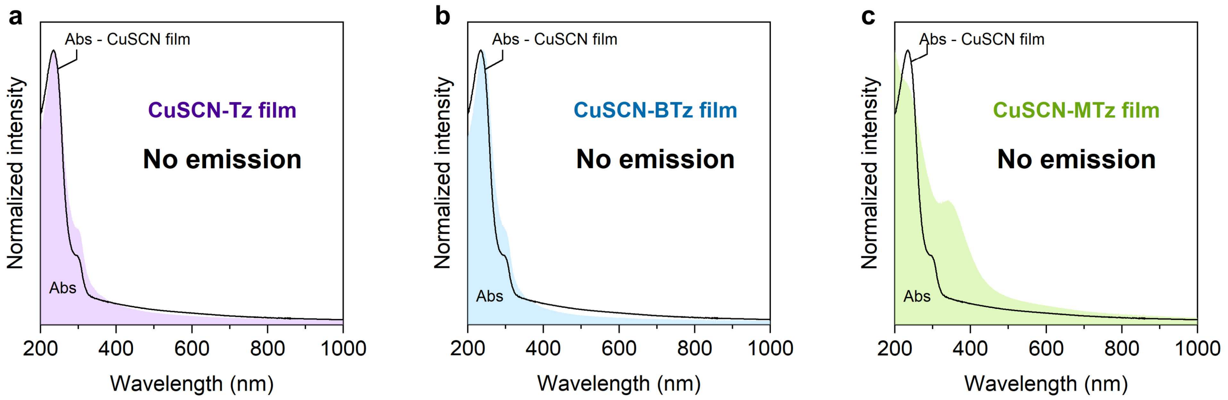

Figure S5. Absorption spectra of (a) CuSCN-Tz, (b) CuSCN-BTz, and (c) CuSCN-MTz in thin-film form. Absorption spectrum of a control CuSCN film is shown as reference in black solid line. For thin films of these three complexes, no photoluminescence was detected.

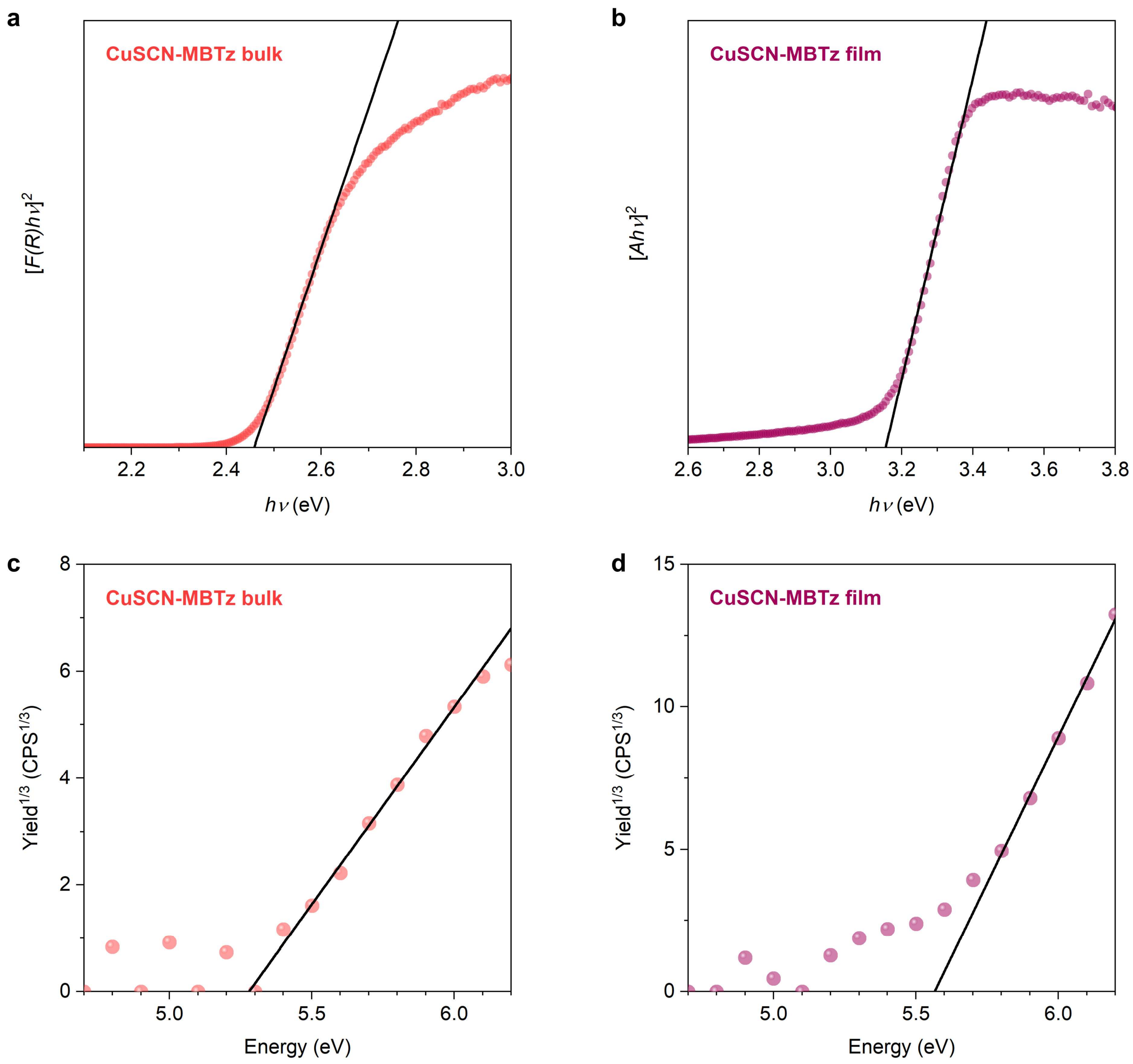

Figure S6. Tauc plots for the determination of the optical band gaps of CuSCN-MBTz in (a) bulk and (b) thin-film forms. For bulk CuSCN-MBTz, the plot was calculated from the Kubelka-Munk function $F(R)$ of the reflectance R. h is the Planck constant, and ν is the photon frequency. Photoemission yield spectra in air for the determination of the highest occupied molecular orbital (HOMO) of CuSCN-MBTz in (c) bulk and (d) thin-film forms.

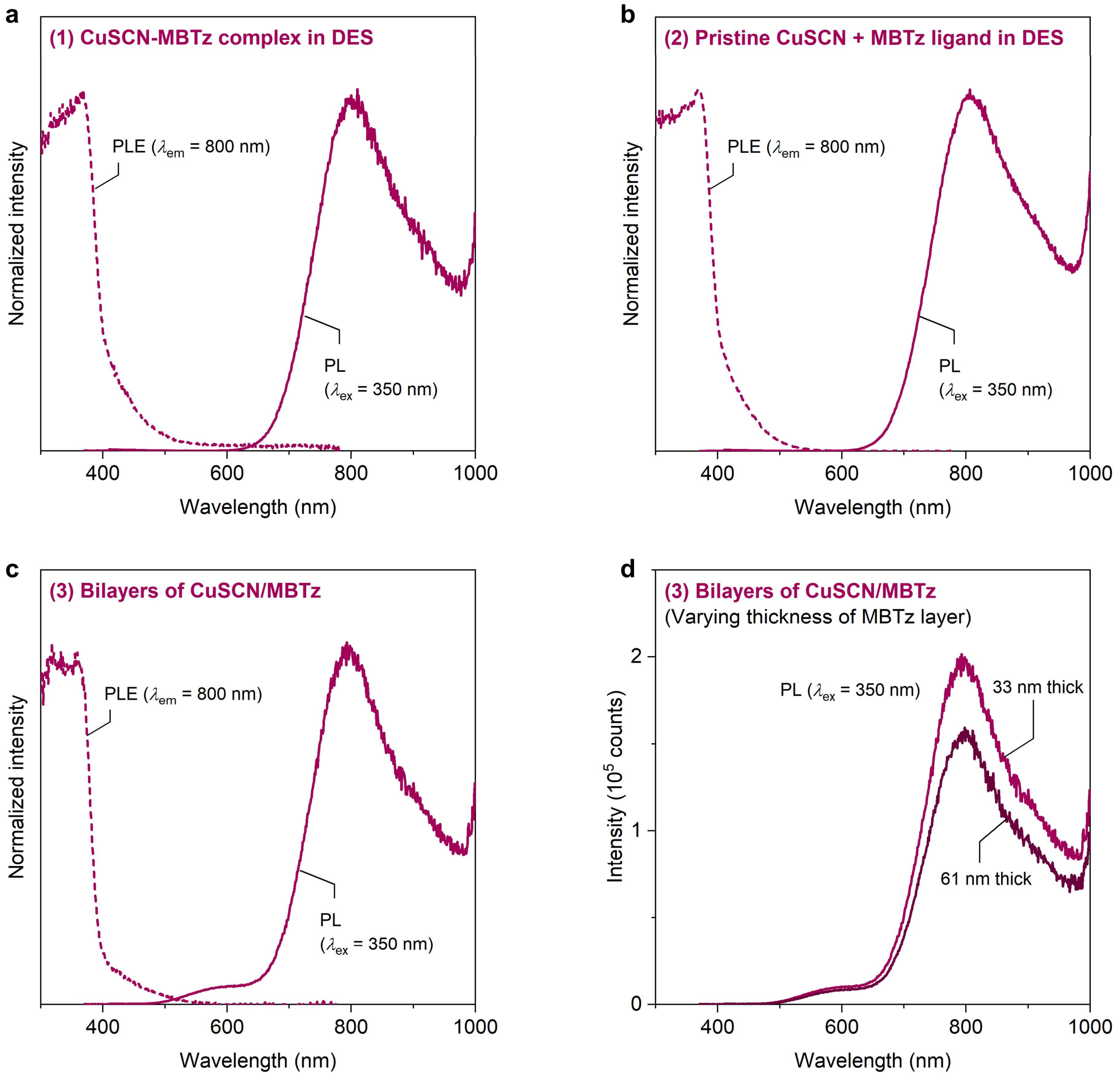

Figure S7. Photoluminescence (PL) spectra and photoluminescence excitation (PLE) spectra of NIR-emitting CuSCN-MBTz films obtained from three different routes: (a) Route (1) by spin-coating a solution of CuSCN-MBTz complex dissolved in DES; (b) Route (2) by spin-coating a solution containing a mixture of pristine CuSCN and pristine MBTz ligand dissolved together in DES; and (c) Route (3) by spin-coating bilayers of pristine CuSCN followed by pristine MBTz ligand. (d) Comparison of two bilayer films also produced by Route (3) but having two different thicknesses of the MBTz layer. Excitation wavelengths (λ_{ex}) and emission wavelengths (λ_{em}) for PL and PLE measurements, respectively, are annotated in the plots.

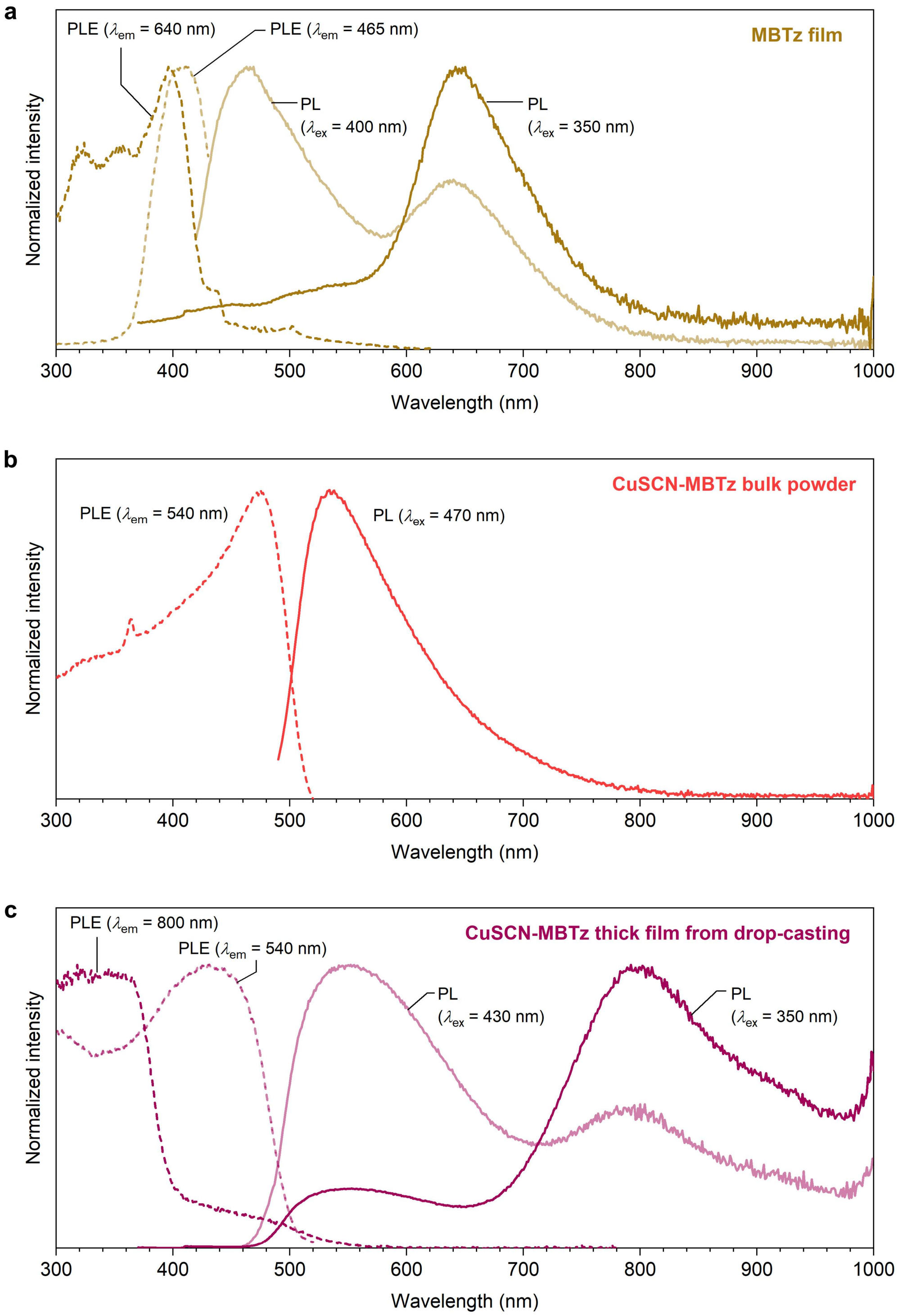

Figure S8. Photoluminescence (PL) spectra and photoluminescence excitation (PLE) spectra of (a) spin-coated film of pristine MBTz, (b) CuSCN-MBTz bulk powder, and (c) CuSCN-MBTz thick film prepared by drop-casting.

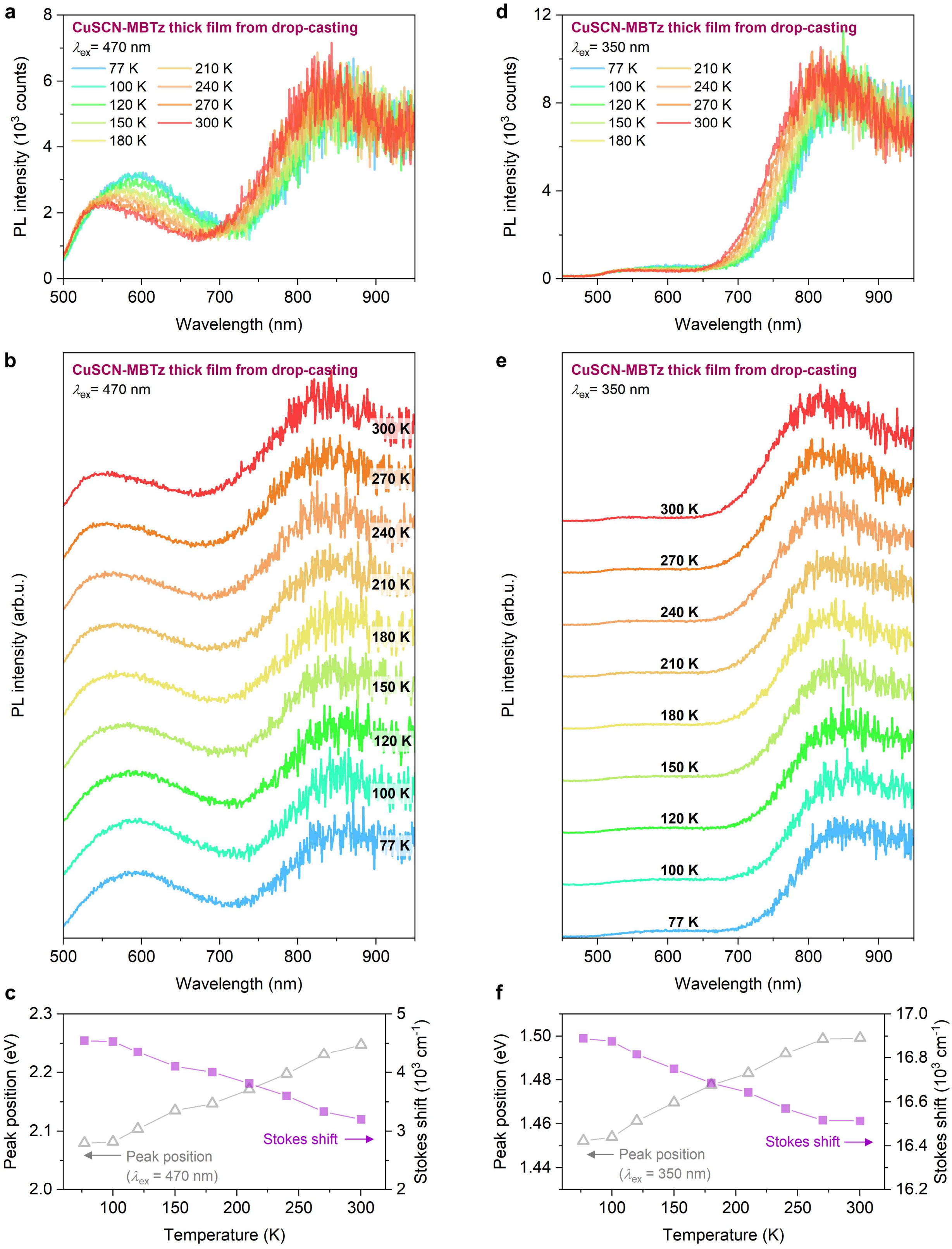

Figure S9. Temperature-dependent photoluminescence (PL) spectroscopy: overlaid plots, stacked plots, and peak position analysis from Gaussian fitting of CuSCN-MBTz thick film from drop-casting, excited at (a-c) 470 nm to observe the higher-energy peak and (d-f) 350 nm to observe the lower energy peak.

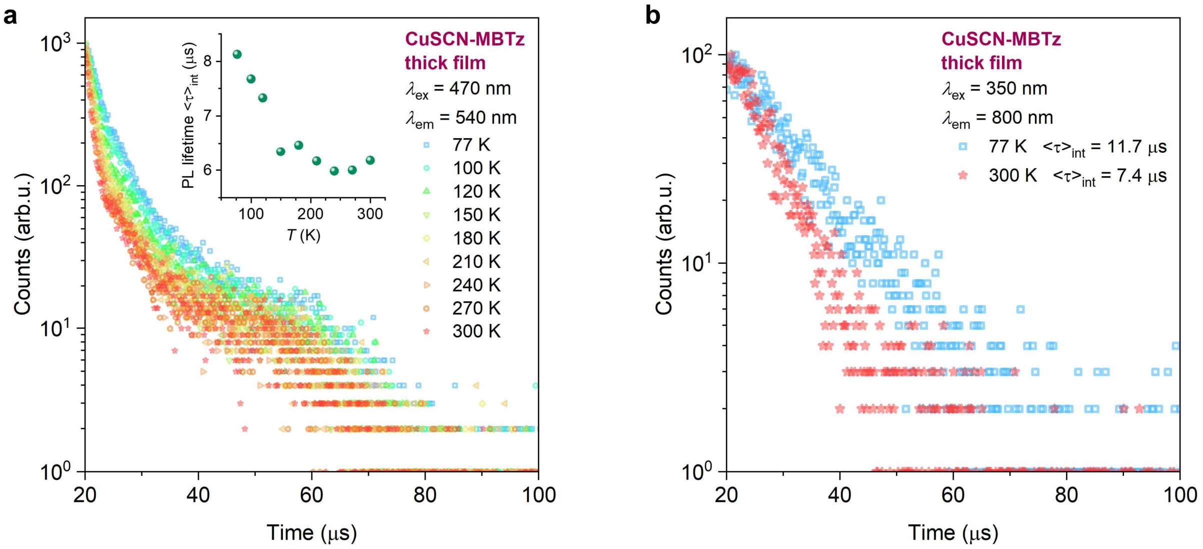

Figure S10. Temperature-dependent time-resolved photoluminescence (TRPL) measurements of CuSCN-MBTz thick film from drop-casting: (a) emission monitored at 540 nm, excited at 470 nm (inset, average PL lifetime); and (b) emission monitored at 800 nm, excited at 350 nm. For (b), due to the low signal in this setup, only data from two temperatures (77 and 300 K) are included to clearly show the change in the decay time (denoted in the legend).

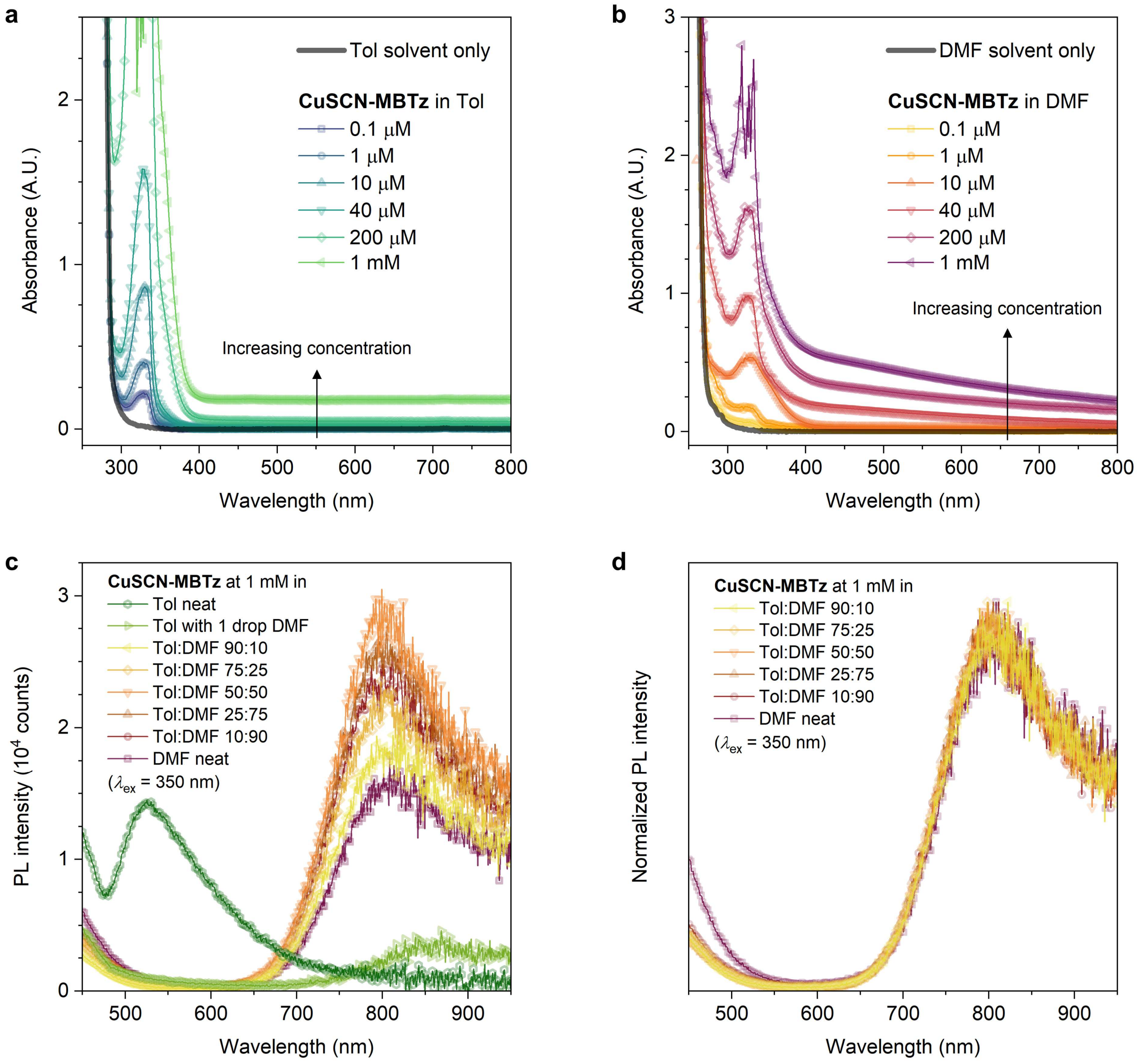

Figure S11. UV-vis absorption spectra of CuSCN-MBTz in (a) toluene (Tol) and (b) dimethylformamide (DMF) at various concentrations. (c) Photoluminescence (PL) spectra of CuSCN-MBTz at 1 mM in Tol:DMF solvent mixtures of various volume ratios. (d) Normalized PL spectra of data from (c).

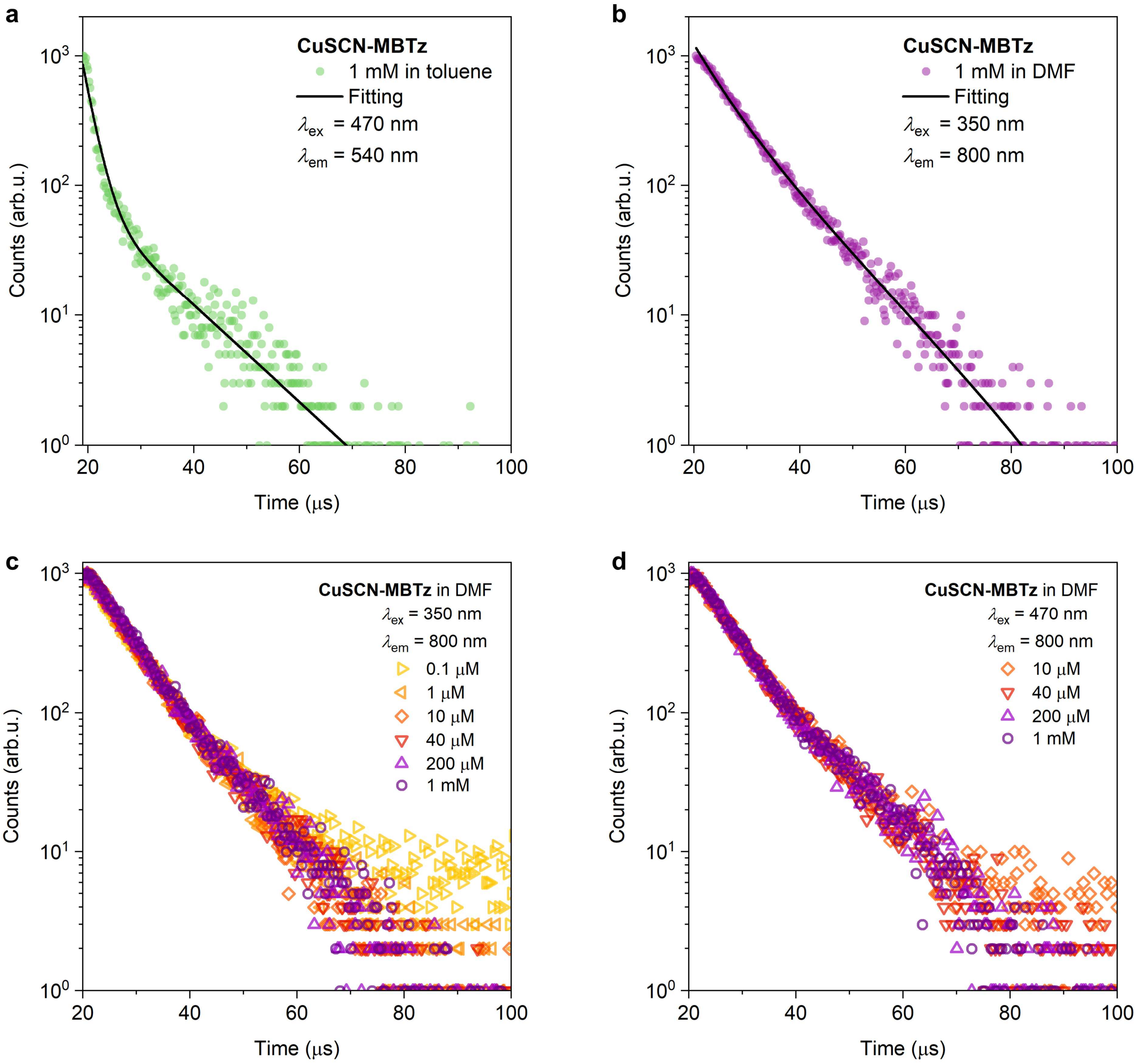

Figure S12. Time-resolved photoluminescence (TRPL) measurements of CuSCN-MBTz at 1 mM in (a) toluene and (b) DMF; and of CuSCN-MBTz at various concentrations in DMF using an excitation source at (c) 350 nm and (d) 470 nm, with the emission at 800 nm monitored for both.

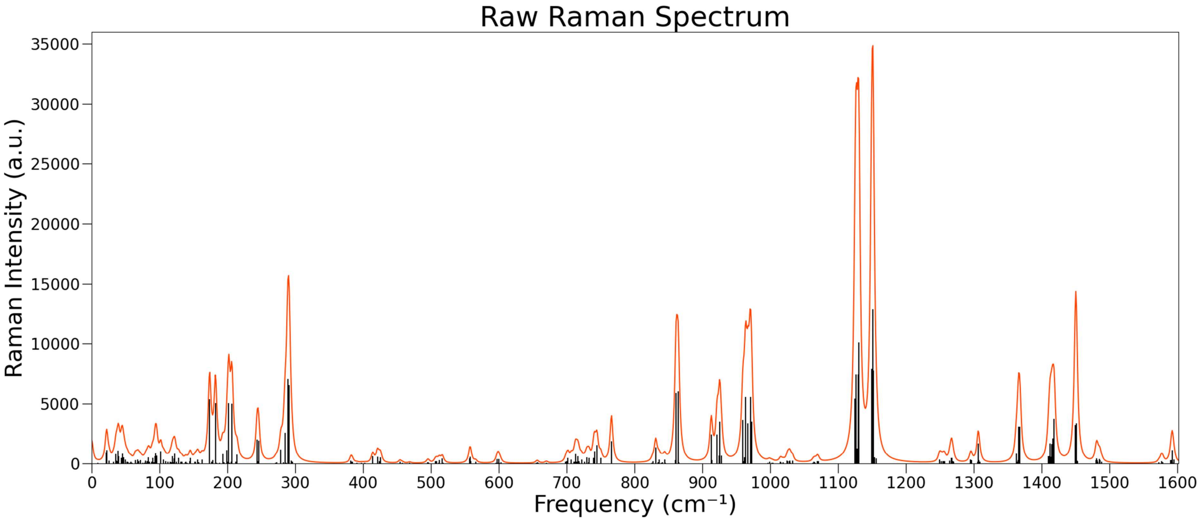

Figure S13. Raw calculated Raman spectrum and transitions before applying a rigid shift.

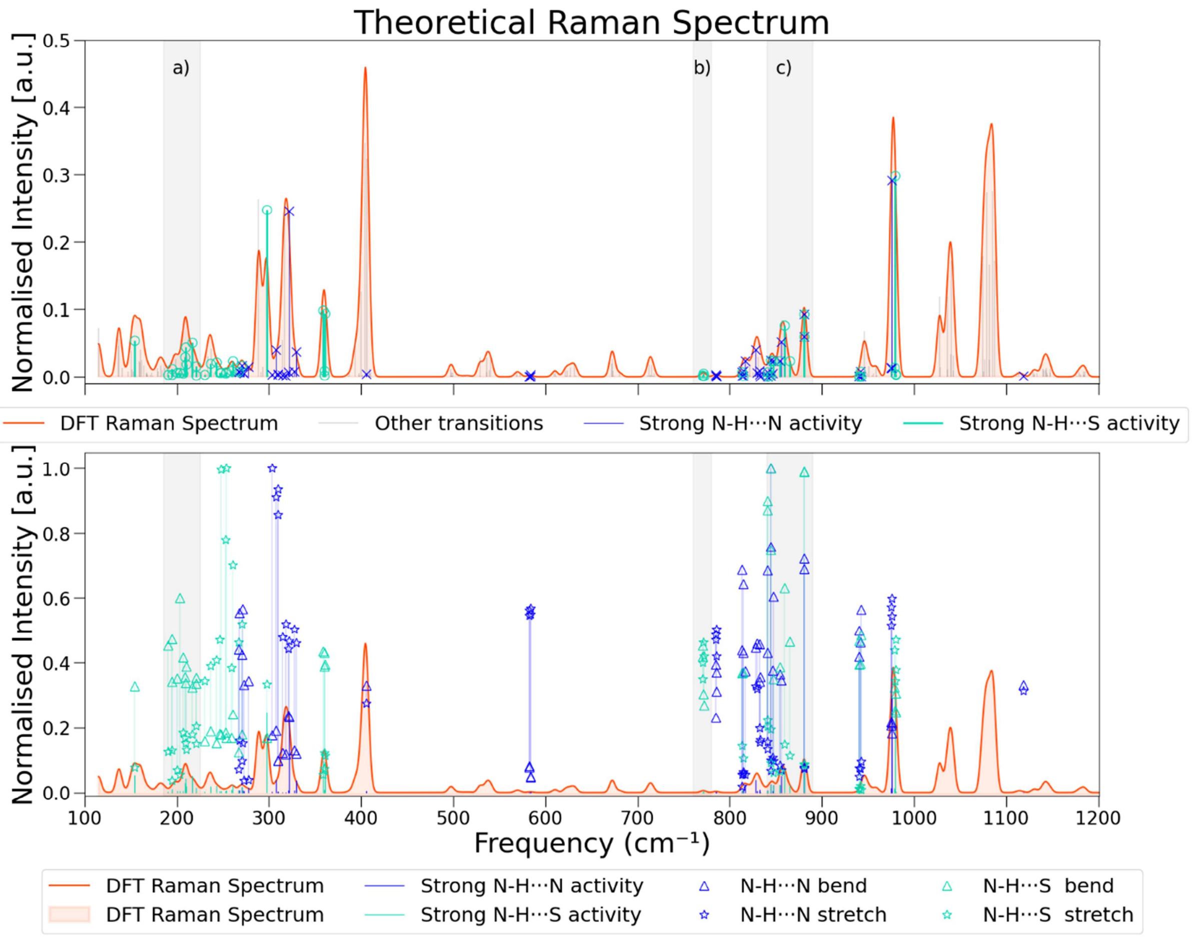

Figure S14. Calculated Raman spectrum and modes with marked intermolecular N–H···S (teal) and intramolecular N–H···N (blue) interactions and their activities.

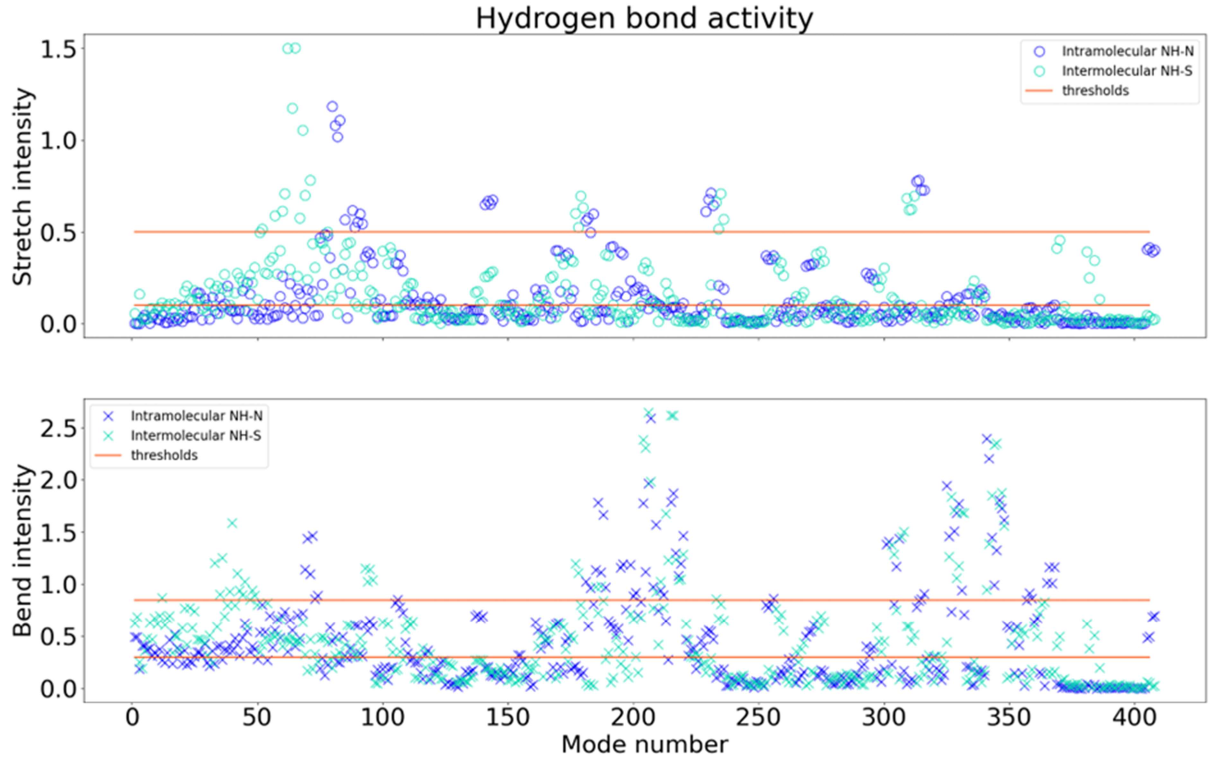

Figure S15. Intensities of (top) stretching and (bottom) bending motions localized to the intermolecular N–H···S (teal) and intramolecular N–H···N (blue) interactions as measured by displacements in the vibrational eigenvectors.

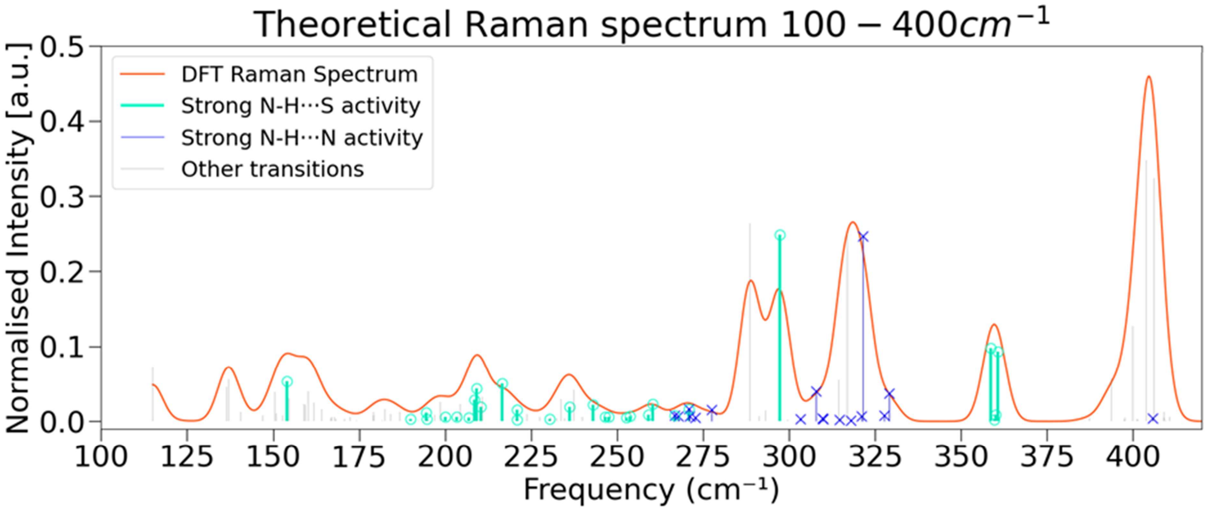

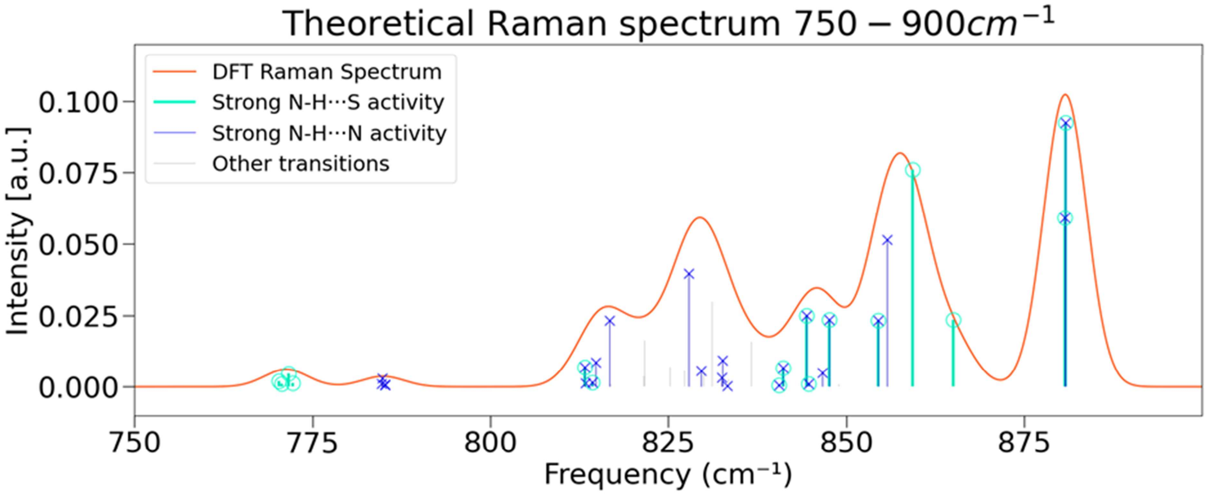

Figure S16. Zoomed in regions of interest in the theoretical Raman spectrum. (Top) The region between 100 – 400 cm^{-1}. (Bottom) The region between 750 – 900 cm^{-1}. These figures more clearly visualize the involvement of intermolecular N–H···S (teal) and intramolecular N–H···N (blue) interactions in the relevant vibrational modes.

Extended discussions on theoretical Raman spectrum

Supplementary File S1 contains a spreadsheet of the spectral data, vibrational mode numbers, and the calculated strength of intermolecular N–H···S and intramolecular N–H···N motions. **Supplementary File S2** contains a .xyz_jmol file with the vibrational animations (open with JMol software, Java required).

Aside from the principal peaks near 206 cm^{-1}, 770 cm^{-1}, and 875 cm^{-1} [**Figure S14** regions (a), (b), and (c)] discussed in the main text, several additional modes in the theoretical spectrum exhibit strong intermolecular activity but are absent from the experimental bulk spectrum. These modes are located at 245 – 275 cm^{-1}, 297 cm^{-1}, 358 – 367 cm^{-1} and 978 – 980 cm^{-1}. These discrepancies most likely reflect limitations of the computational methodology and indicate that the calculated Raman intensities should be treated as qualitative rather than quantitatively accurate. Some of these locations (297 cm^{-1} and 978 – 980 cm^{-1}) may be interesting from the perspective of comparing bulk and film, but the issues in both the film and theoretical spectra makes comparison infeasible.

In general, the low-frequency modes (approx. 115-500 cm^{-1}, modes 1-100) exhibit predominantly intermolecular character, while the mid-frequency range (approx. 500-1350 cm^{-1}, modes 100-300) consists of mixed intra- and intermolecular vibrations, with intermolecular coupling strongest at the lower end. High-frequency modes (approx. >1350 cm^{-1}, modes >300) are largely intramolecular in nature.

Regarding the possible candidates for the bulk spectrum peak at 795 cm^{-1}. The bands around 770 cm^{-1} arise from intermolecular N–H···S active vibrations (modes 177-180). These vibrations are characterized by coupled thiazole ring stretching and N–H wagging vibrations with intermolecular coupling via the N–H···S interaction.

The peaks between 800-900 cm^{-1} feature a mixture of both intermolecular N–H···S and intramolecular N–H···N activity. Modes 185 and 187 (at 813 cm^{-1}) closely resemble modes 177–180, composed of coupled thiazole ring stretching and N–H wagging vibrations with intermolecular coupling. Modes 204-209 and 211-214 – key components of the peaks between 840-860 cm^{-1} – are dominated by collective hydrogen wagging motions, involving both the N–H···S and N–H···N dipole-dipole interactions. These collective hydrogen motions become increasingly localized as the frequency increases. Finally, the modes 215, and 216 at 880 cm^{-1} are N–H wagging vibrations almost entirely localized on both N–H···S and N–H···N dipole-dipole interactions. Note, mode 210 features very low dipole-dipole activity, as the hydrogen wagging is strongly localized to the benzene rings.

It is notable that the most intense Raman peaks with strong intermolecular H-bond-like activity originate from bending rather than stretching (**Figure S14**). This is expected, as bending modes of H-interactions generally induce greater changes to the polarizability tensor, and hence possess greater Raman activity. Conversely, stretching modes modulate the dipole moment and are thereby more IR active.

It is also interesting that the peaks at 320 cm^{-1} (293 cm^{-1} exp.), and 975 cm^{-1} (1015 cm^{-1} exp.) involving strong intramolecular motions alone do not seem to be strongly affected in the thin film. This suggests intramolecular H-interactions remain in the film, whilst intermolecular H-interactions are disrupted. We make

this point tentatively, as the thin-film spectrum is relatively noisy and hampers precise comparison. However, it is further supported by DFT calculations, which show a shorter inter-atomic distance of the intramolecular N–H···N interaction in the molecular geometry (1.99 Å) compared to bulk (2.18 Å).

References

(1) Li, M.; Li, B.; Chen, J.; Shen, X.; Cui, S.; He, X.; Liu, K.; Han, Q. Analysis of Thermal Decomposition of Acidified Sediments in Gold Plants and Harmless Disposal of It. *J. Hazard. Mater.* **2022**, *431* (December 2021), 128472. https://doi.org/10.1016/j.jhazmat.2022.128472.

(2) Kabešová, M.; Šramko, T.; Gažo, J.; Zumadilov, E. K.; Nefedov, V. I. Thermal Properties of Thiocyanatocopper(II) Complexes with Picolines and Lutidines. *J. Therm. Anal.* **1978**, *13* (1), 55–64. https://doi.org/10.1007/BF01909908.

(3) Waldner, P. Solid-State Phase Equilibria of the Cu-S System: Thermodynamic Modeling. *J. Phase Equilibria Diffus.* **2018**, *39* (6), 810–819. https://doi.org/10.1007/s11669-018-0670-z.
